# Characterizing rare and low-frequency height-associated variants in the Japanese population

**DOI:** 10.1038/s41467-019-12276-5

**Published:** 2019-09-27

**Authors:** Masato Akiyama, Kazuyoshi Ishigaki, Saori Sakaue, Yukihide Momozawa, Momoko Horikoshi, Makoto Hirata, Koichi Matsuda, Shiro Ikegawa, Atsushi Takahashi, Masahiro Kanai, Sadao Suzuki, Daisuke Matsui, Mariko Naito, Taiki Yamaji, Motoki Iwasaki, Norie Sawada, Kozo Tanno, Makoto Sasaki, Atsushi Hozawa, Naoko Minegishi, Kenji Wakai, Shoichiro Tsugane, Atsushi Shimizu, Masayuki Yamamoto, Yukinori Okada, Yoshinori Murakami, Michiaki Kubo, Yoichiro Kamatani

**Affiliations:** 1 Laboratory for Statistical Analysis, RIKEN Center for Integrative Medical Sciences, Yokohama, 230-0045 Japan; 2 Department of Ophthalmology, Graduate School of Medical Sciences, Kyushu University, Fukuoka, 812-8582 Japan; 3 Laboratory for Statistical and Translational Genetics, RIKEN Center for Integrative Medical Sciences, Yokohama, 230-0045 Japan; 4 Department of Allergy and Rheumatology, Graduate School of Medicine, The University of Tokyo, Tokyo, 113-8655 Japan; 5 Laboratory for Genotyping Development, RIKEN Center for Integrative Medical Sciences, Yokohama, 230-0045 Japan; 6 Laboratory for Endocrinology, Metabolism and Kidney Diseases, RIKEN Center for Integrative Medical Sciences, Yokohama, 230-0045 Japan; 7 Laboratory of Genome Technology, Human Genome Center, Institute of Medical Science, The University of Tokyo, Tokyo, 108-8639 Japan; 8 Laboratory of Clinical Genome Sequencing, Department of Computational Biology and Medical Sciences, Graduate school of Frontier Sciences, The University of Tokyo, Tokyo, 108-8639 Japan; 9 Laboratory for Bone and Joint Diseases, RIKEN Center for Integrative Medical Sciences, Tokyo, 108-8639 Japan; 10 Department of Genomic Medicine, Research Institute, National Cerebral and Cardiovascular Center, Osaka, 565-8565 Japan; 11 Department of Biomedical Informatics, Harvard Medical School, Boston, MA02115 USA; 12 Nagoya City University Graduate School of Medical Sciences, Nagoya, 467-8601 Japan; 13 Department of Epidemiology for Community Health and Medicine, Kyoto Prefectural University of Medicine, Graduate School of Medical Science, Kyoto, 602-8566 Japan; 14 Department of Preventive Medicine, Nagoya University Graduate School of Medicine, Nagoya, 466-8550 Japan; 15 Department of Oral Epidemiology, Graduate School of Biomedical and Health Sciences, Hiroshima University, Hiroshima, 734-8553 Japan; 16 Division of Epidemiology, Center for Public Health Sciences, National Cancer Center, Tokyo, 104-0045 Japan; 17 Iwate Tohoku Medical Megabank Organization, Iwate Medical University, Iwate, 028-3694 Japan; 18 Department of Hygiene and Preventive Medicine, School of Medicine, Iwate Medical University, Iwate, 028-3694 Japan; 19 Tohoku Medical Megabank Organization, Tohoku University, Sendai, 980-8573 Japan; 20 Graduate School of Medicine, Tohoku University, Sendai, 980-8575 Japan; 21 Center for Public Health Sciences, National Cancer Center, Tokyo, 104-0045 Japan; 22 Department of Statistical Genetics, Osaka University Graduate School of Medicine, Osaka, 565-0871 Japan; 23 Laboratory of Statistical Immunology, Immunology Frontier Research Center (WPI-IFReC), Osaka University, Osaka, 565-0871 Japan; 24 Division of Molecular Pathology, The Institute of Medical Science, The University of Tokyo, Tokyo, 108-8639 Japan; 25 RIKEN Center for Integrative Medical Sciences, Yokohama, 230-0045 Japan; 26 Laboratory of Complex Trait Genomics, Department of Computational Biology and Medical Sciences, Graduate School of Frontier Sciences, The University of Tokyo, Tokyo, 108-8639 Japan

**Keywords:** Genome-wide association studies, Heritable quantitative trait, Rare variants, Quantitative trait

## Abstract

Human height is a representative phenotype to elucidate genetic architecture. However, the majority of large studies have been performed in European population. To investigate the rare and low-frequency variants associated with height, we construct a reference panel (*N* = 3,541) for genotype imputation by integrating the whole-genome sequence data from 1,037 Japanese with that of the 1000 Genomes Project, and perform a genome-wide association study in 191,787 Japanese. We report 573 height-associated variants, including 22 rare and 42 low-frequency variants. These 64 variants explain 1.7% of the phenotypic variance. Furthermore, a gene-based analysis identifies two genes with multiple height-increasing rare and low-frequency nonsynonymous variants (*SLC27A3* and *CYP26B1*; *P*_SKAT-O_ < 2.5 × 10^−6^). Our analysis shows a general tendency of the effect sizes of rare variants towards increasing height, which is contrary to findings among Europeans, suggesting that height-associated rare variants are under different selection pressure in Japanese and European populations.

## Abstract (web summary)

Thousands of genetic loci are known to associate with human height, but these are mainly based on studies in European ancestry populations. Here, Akiyama et al. construct a genotype reference panel for the Japanese population followed by GWAS and report 573 height associated variants in 191,787 Japanese.

## Introduction

Human height is a highly heritable trait under polygenic inheritance^1^. Over the past decade, genome-wide association studies (GWAS) have identified more than 3500 variants associated with human height^2–8^. Furthermore, recent studies using high-density genotype imputation based on the whole-genome sequencing data^4–6^ and commercial genotyping array focused on coding variants^7^ and uncovered dozens of rare and low-frequency variants with greater impacts on human height. Meanwhile, investigations in non-European populations have been performed with relatively smaller sample sizes^3^. Therefore, there were limited variants reportedly associated with height in non-European population, such as East Asians. Considering that diverse populations harbor ancestral-specific rare variants^9^, searching for such genetic variations in East Asian population with large samples would expand our knowledge on genetic components associated with complex traits.

Here we report a large-scale association study (*N* > 190,000) of human height in a Japanese population using a newly constructed reference panel for genotype imputation, including deep whole-genome sequence (WGS) data of 1037 Japanese^10^. Our primary aim of this study is to identify rare (minor allele frequency (MAF) <1%) and low-frequency (1% ≤ MAF < 5%) variants associated with height in the Japanese population. To characterize the roles of the identified variants, we further evaluate the gene-level associations and pleiotropic effects of the identified genes and variants, and investigate the natural selection on height-associated rare variants. Through the study, we identify 573 variants associated with height in the Japanese population. Of these, 22 are rare and 42 are low-frequency variants. We also reveal two height-associated genes that have not been reported. Moreover, our analysis of selection on height-associated variants suggests different selection pressure between Japanese and European populations.

## Results

### Reference panel for genotype imputation

**Fig. 1:**
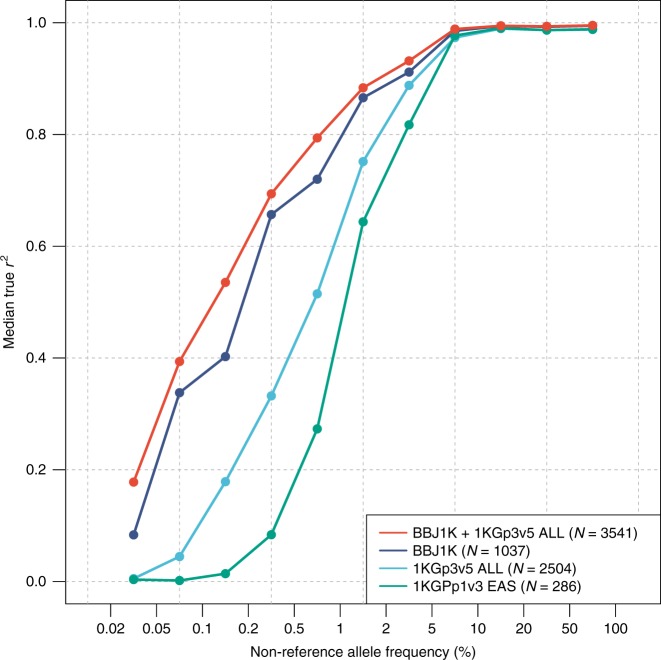
Plot of imputation qualities by different reference panels. The accuracy of imputation was evaluated for four different reference panels at “masked” HumanExome SNPs. BBJ1K + 1KGp3v5 panel (red) showed the best performance at every allele frequency bins. Even the BBJ1K panel showed much better accuracy than the 1KGp3v5 ALL panel regardless of the difference in sample size, indicating the contribution of deep haplotype information of the specific population to imputation accuracy. Median *r*^2^ values are shown on the *y*-axis

The study design is illustrated in Supplementary Fig. 1. To achieve a better imputation accuracy for rare and low-frequency variants, we constructed a reference panel (*N* = 3541) composed of deep WGS data obtained from 1037 Japanese individuals^10^ (BBJ1K; Supplementary Note 1) and the 1000 Genomes Project^9^ (1KG; phase3v5, *N* = 2504). We compared the imputation accuracy with that of other reference panels and found that the integrated panel exhibited the best imputation accuracy, especially for rare and low-frequency variants (Fig. 1 and Supplementary Fig. 2).

### Genome-wide association study

After the application of stringent quality control (QC) and whole-genome imputation to the new reference panel, we conducted a GWAS in 159,195 participants of the Biobank Japan (BBJ) project^11,12^ using 27,896,057 imputed variants (Supplementary Fig. 3a and Supplementary Table 1). As expected, we observed high genomic inflation (*λ*_GC_ = 1.20 for all variants and 1.69 for variants with MAF ≥1%; Supplementary Fig. 3b). Since we used a linear mixed model^13^ to test association, we interpreted this inflation as being due to polygenic effects. We quantified the bias resulting from population stratification and cryptic relatedness using linkage disquilibrium (LD) score regression^14^. The mean *χ*^2^ value was 2.63, intercept was 1.21 ± 0.02 (mean ± standard error [s.e.]) and ratio was 0.13. This proportion was comparable with those in our previous GWASs for quantitative traits in the BBJ^15,16^.

We defined genetic loci by considering the positional relationships of the variants reached genome-wide significance (*P*_GWAS_ <5.0 × 10^−8^; Supplementary Fig. 4a). If the significantly associated variants at two different loci were more than 1 Mb apart from each other, we regarded these loci as independent. As a result, we observed 363 independent loci, including nine novel loci with genome-wide significance (Supplementary Data 1, 2 and Supplementary Table 2).

Next, to investigate independently associated signals underlying the identified 363 loci, we performed several rounds of conditional analyses by repeating the association analysis using BOLT-LMM until the association of the top variants fell below the significance threshold. This analysis revealed an additional 246 independently associated variants satisfying the genome-wide significance within 130 loci (Supplementary Data 2, 3 and Supplementary Fig. 5). In total, 609 lead variants reached a genome-wide significant level in the GWAS. We identified a high number of independently associated signals near *ACAN* (*N*_signal_ = 7), *ADAMTS17* (*N*_signal_ = 11), and *IGFBP5*-*IHH*-*RESP18* (*N*_signal_ = 10), which were also observed in a study conducted in a European population^2^. When we examined the frequencies of the identified variants in the 1KG (Supplementary Data 4 and Supplementary Fig. 6), 40 were found to be monomorphic in all non-East Asian populations, suggesting that these variants are East Asian specific.

**Fig. 2:**
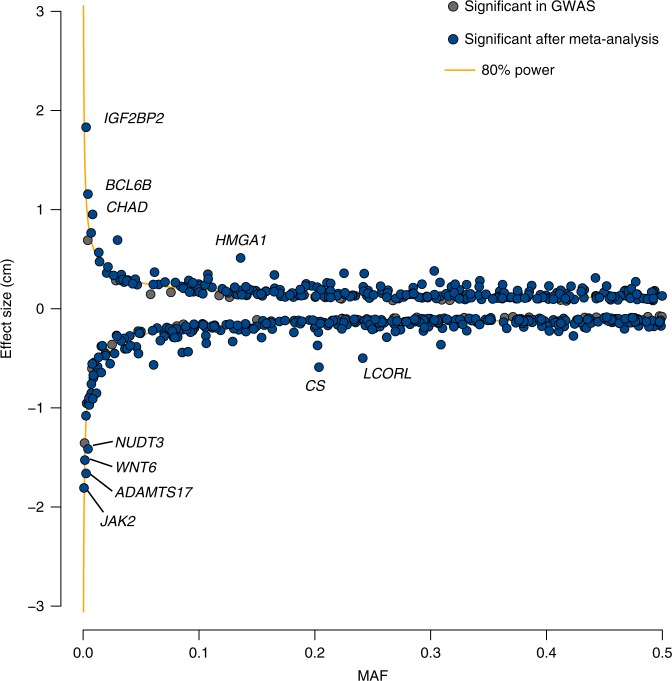
Scatter plot of MAF and effect size. We plotted the allele frequency (*x-*axis) and effect size (*y*-axis) of the minor alleles of the 609 variants that reached genome-wide significance in the discovery GWAS. Variants that remained genome-wide significant after the meta-analysis are indicated in blue. The orange line denotes 80% power to achieve genome-wide significance. To convert the standardized effect size to a centimeter scale, we regarded 1 standard error as 5.63 cm, which was calculated in the population-based cohorts (replication set)

We evaluated 609 identified variants in 32,692 independent individuals as a replication study (Supplementary Note 2, Supplementary Table 1, and Supplementary Data 5). Among these variants, 415 (68.1%) were nominally associated (*P*_rep_ < 0.05) in the same direction of effects, and 598 variants (98.2%) showed directional consistency (*P* for sign test = 9.38 × 10^−161^), suggesting that the majority of the identified variants were not false positives. We also observed a strong correlation of the effect sizes between the GWAS and replication (Pearson’s *r* = 0.88, *P* = 3.47 × 10^−194^, Supplementary Fig. 7). After a fixed-effect meta-analysis, 573 variants (95.5%) remained genome-wide significant (Supplementary Data 5) and we determined that these were significantly associated throughout the present study. Of these variants, 124 (21.6%) were replicated with Bonferroni-corrected level of associations (*α* = 8.21 × 10^−5^ [=0.05/609]) and 289 (50.4%) were replicated with nominal significance (8.21 × 10^−5^ < *P*_rep_ < 0.05). We did not observe Bonferroni-corrected level of heterogeneity of effect sizes between GWAS and the replication study (*P*_het_ > 8.21 × 10^−5^). Among the 573 variants, 22 were rare and 42 were low-frequency variants. The effect sizes of the rare and low-frequency variants showed stronger effects than those of common variants (Fig. 2). The rare and low-frequency variants that remained genome-wide significant after the meta-analysis (*N* = 64) together explained 1.7% of the phenotypic variance in the replication set (Tables 1 and 2), which was comparable to findings in the European population (1.7% by 83 variants)^7^.

**Table 1:** Height-associated rare variants in the Japanese population

Chr:Pos_REF/ALT	Candidate genes (annotation)	ALT freq. (%)	*β* (SE) in GWAS	*P*_GWAS_	*β* (SE) in Rep	*P*_Rep_	VE (%)	*β* (SE) in meta	*P*_meta_
1:17,2107,982_G/A	*DNM3OS*, *MIR214*, *MIR3120*	0.25	−0.195 (0.033)	1.4 × 10^−^^10^	−0.167 (0.089)	8.9 × 10^−^^2^	0.010	−0.192 (0.031)	4.5 × 10^−^^10^
2:1,638,793_C/T	*PXDN*	0.61	−0.163 (0.021)	6.5 × 10^−^^17^	−0.129 (0.045)	1.9 × 10^−^^3^	0.022	−0.157 (0.019)	5.5 × 10^−^^17^
2:219,729,259_G/A	*WNT6* (EAS)	0.15	−0.278 (0.051)	8.7 × 10^−^^9^	−0.241 (0.110)	4.4 × 10^−^^2^	0.019	−0.271 (0.046)	4.0 × 10^−^^9^
2:219,919,943_C/T	*IHH* (NS, EP, EAS, PE)	0.30	−0.177 (0.029)	8.4 × 10^−^^11^	−0.127 (0.070)	4.9 × 10^−^^2^	0.009	−0.170 (0.027)	1.9 × 10^−^^10^
3:52,781,335_G/A	*NEK4*	0.90	−0.118 (0.018)	6.9 × 10^−^^11^	−0.152 (0.043)	2.4 × 10^−^^4^	0.039	−0.123 (0.016)	8.4 × 10^−^^14^
3:145,794,588_T/C	*PLOD2* (NS, EP, EAS)	0.69	0.131 (0.021)	3.8 × 10^−^^11^	0.164 (0.050)	8.3 × 10^−^^4^	0.035	0.136 (0.019)	1.4 × 10^−^^12^
3:185,513,482_A/G	*IGF2BP2*	0.26	0.384 (0.053)	3.7 × 10^−^^13^	0.120 (0.099)	2.1 × 10^−^^1^	0.009	0.325 (0.047)	3.4 × 10^−^^12^
4:17,880,391_T/A	*LCORL* (EAS)	0.88	−0.135 (0.021)	9.9 × 10^−^^12^	−0.068 (0.047)	9.8 × 10^−^^2^	0.008	−0.124 (0.019)	6.9 × 10^−^^11^
6:33,096,707_T/C	*HLA-DPB2* (EAS)	0.93	−0.106 (0.018)	1.8 × 10^−^^8^	−0.050 (0.040)	4.1 × 10^−^^1^	0.005	−0.097 (0.017)	7.4 × 10^−^^9^
6:34,257,615_C/A	*NUDT3*, *RPS10-NUDT3* (EAS)	0.42	−0.245 (0.027)	3.3 × 10^−^^20^	−0.283 (0.061)	1.3 × 10^−^^6^	0.070	−0.251 (0.025)	8.5 × 10^−^^24^
7:45,954,540_C/T	*IGFBP3* (NS, EAS)	0.70	−0.153 (0.021)	3.5 × 10^−^^14^	−0.142 (0.053)	5.2 × 10^−^^3^	0.026	−0.151 (0.019)	7.4 × 10^−^^15^
8:57,283,748_G/A	*SDR16C5*, *SDR16C6P*	0.46	−0.161 (0.026)	9.4 × 10^−^^11^	−0.194 (0.062)	3.7 × 10^−^^4^	0.032	−0.166 (0.024)	6.8 × 10^−^^12^
9:5,089,770_G/A	*JAK2* (NS, EAS, PE)	0.08	−0.344 (0.060)	1.1 × 10^−^^8^	−0.190 (0.146)	2.0 × 10^−^^1^	0.005	−0.321 (0.056)	8.5 × 10^−^^9^
9:95,123,569_C/T	*CENPP*	0.54	−0.177 (0.024)	2.9 × 10^−^^15^	−0.150 (0.055)	8.1 × 10^−^^3^	0.025	−0.172 (0.022)	3.7 × 10^−^^15^
12:2,973,887_C/A	*FOXM1* (NS, EAS)	0.78	−0.115 (0.017)	7.2 × 10^−^^11^	−0.005 (0.042)	9.2 × 10^−^^1^	0.000	−0.099 (0.016)	8.5 × 10^−^^10^
15:85574,143_T/A	*PDE8A* (NS, EAS)	0.50	−0.149 (0.023)	2.3 × 10^−^^11^	−0.224 (0.055)	5.8 × 10^−^^5^	0.045	−0.160 (0.021)	2.1 × 10^−^^14^
15:89,438,963_ACG/A	*HAPLN3*	0.88	−0.127 (0.019)	1.2 × 10^−^^10^	−0.086 (0.040)	3.0 × 10^−^^2^	0.015	−0.119 (0.017)	4.2 × 10^−^^12^
15:89,459,676_G/A	*MFGE8*, *ABHD2*	0.71	−0.128 (0.021)	2.5 × 10^−^^9^	−0.165 (0.045)	1.1 × 10^−^^3^	0.043	−0.135 (0.019)	1.6 × 10^−^^12^
15:100,533,312_C/T	*ADAMTS17* (NS, EP, EAS)	0.25	−0.275 (0.036)	1.3 × 10^−^^14^	−0.398 (0.081)	2.8 × 10^−^^7^	0.078	−0.295 (0.033)	2.5 × 10^−^^19^
17:6,925,490_C/T	*BCL6B*	0.42	0.203 (0.026)	4.3 × 10^−^^15^	0.219 (0.057)	3.6 × 10^−^^4^	0.045	0.206 (0.024)	9.5 × 10^−^^18^
17:48,545,926_C/A	*CHAD* (NS, EAS)	0.82	0.169 (0.018)	9.8 × 10^−^^22^	0.171 (0.041)	7.9 × 10^−^^6^	0.045	0.169 (0.016)	2.2 × 10^−^^25^
17:61,976,011_G/T	*GH1* (NS, EP)	0.83	−0.154 (0.018)	5.4 × 10^−^^18^	−0.202 (0.043)	2.4 × 10^−^^6^	0.065	−0.162 (0.017)	5.0 × 10^−^^22^

Results of the association analysis in GWAS and replication (Rep). Variants with minor allele frequency <1% satisfying genome-wide significance after meta-analysis were shown. Annotation: EAS, East Asian specific variant determined by allele frequency in 1000 Genomes Project; NS, variant is in LD with nonsynonymous variant(s); NL, variant was found in new loci identified by this study; EP, gene is associated with extremely phenotype related to height. Chromosomes and positions are based on Build37 (hg19)

*REF* reference allele, *ALT* alternative allele, *SE* standard error, *VE* variance explained

**Table 2:** Height-associated low-frequency variants in the Japanese population

Chr:Pos_REF/ALT	Candidate genes (annotation)	ALT freq. (%)	*β* (SE) in GWAS	*P*_GWAS_	*β* (SE) in Rep	*P*_Rep_	VE (%)	*β* (SE) in meta	*P*_meta_
1:119,482,365_T/TA	*TBX15* (EAS)	4.02	−0.051 (0.008)	3.4 × 10^−^^10^	−0.035 (0.019)	8.7 × 10^−^^2^	0.010	−0.049 (0.008)	1.5 × 10^−^^10^
1:151,035,628_A/C	*MLLT11*	3.19	0.051 (0.009)	1.1 × 10^−^ ^9^	0.052 (0.021)	1.2 × 10^−^^2^	0.016	0.051 (0.008)	2.2 × 10^−^^10^
1:153,750,682_G/A	*SLC27A3* (NS, MS)	1.42	0.092 (0.013)	1.5 × 10^−^^12^	0.039 (0.031)	3.7 × 10^−^^1^	0.004	0.084 (0.012)	1.9 × 10^−^^12^
1:218,698,632_CG/C	*MIR548F3*	3.85	0.051 (0.008)	1.4 × 10^−^^10^	0.053 (0.019)	6.6 × 10^−^^3^	0.021	0.052 (0.007)	3.2 × 10^−^^12^
2:240,77,979_G/A	*ATAD2B* (EAS)	3.54	−0.048 (0.009)	8.2 × 10^−^^10^	−0.076 (0.019)	5.5 × 10^−^^5^	0.044	−0.053 (0.008)	9.0 × 10^−^^12^
2:62,191,772_A/G	*COMMD1*	4.50	0.044 (0.008)	7.9 × 10^−^^9^	0.065 (0.018)	7.0 × 10^−^^4^	0.035	0.047 (0.007)	1.7 × 10^−^^11^
2:72,360,160_C/T	*CYP26B1* (NS, EP, EAS, MS)	3.30	0.059 (0.009)	6.9 × 10^−^^13^	0.073 (0.021)	4.3 × 10^−^^4^	0.030	0.061 (0.008)	3.5 × 10^−^^14^
2:72,572,735_T/C	*EXOC6B* (EAS)	2.97	0.122 (0.010)	8.9 × 10^−^^32^	0.131 (0.025)	1.2 × 10^−^^6^	0.086	0.123 (0.010)	7.4 × 10^−^^38^
2:74,781,375_C/G	*DOK1*	1.25	−0.114 (0.015)	3.6 × 10^−^^14^	−0.053 (0.034)	1.1 × 10^−^^1^	0.007	−0.104 (0.014)	6.2 × 10^−^^14^
2:95,336,090_G/T	*NONE*, *FAM95A*	2.33	−0.107 (0.017)	2.2 × 10^−^^10^	−0.066 (0.034)	2.6 × 10^−^^2^	0.022	−0.099 (0.015)	8.2 × 10^−^^11^
2:200,141,875_G/A	*SATB2*	2.71	−0.076 (0.010)	1.2 × 10^−^^13^	−0.100 (0.022)	1.8 × 10^−^^5^	0.058	−0.080 (0.009)	6.7 × 10^−^^18^
2:21,9894,893_T/A	*CCDC108* (NS, EAS)	1.93	−0.080 (0.012)	1.6 × 10^−^^12^	−0.081 (0.026)	2.1 × 10^−^^3^	0.027	−0.080 (0.011)	4.4 × 10^−^^14^
2:220,210,184_G/A	*RESP18*, *DNPEP* (EAS)	2.03	0.062 (0.012)	3.8 × 10^−^^8^	0.076 (0.026)	6.2 × 10^−^^3^	0.024	0.064 (0.011)	1.6 × 10^−^^9^
2:241,783,039_C/A	*KIF1A*, *AGXT* (EAS)	4.67	0.045 (0.008)	7.9 × 10^−^^10^	0.031 (0.018)	1.0 × 10^−^^1^	0.008	0.043 (0.007)	4.6 × 10^−^^10^
4:17,880,472_T/A	*LCORL*	3.60	−0.067 (0.010)	1.4 × 10^−^^11^	−0.096 (0.023)	8.5 × 10^−^^5^	0.063	−0.072 (0.009)	1.3 × 10^−^^14^
6:34,2409,03_C/T	*C6orf1*, *NUDT3* (EAS)	1.35	0.086 (0.015)	1.5 × 10^−^^9^	0.172 (0.033)	1.0 × 10^−^^7^	0.084	0.101 (0.013)	7.2 × 10^−^^14^
6:35,243,836_C/T	*ZNF76*	4.95	−0.045 (0.007)	2.2 × 10^−^^10^	−0.041 (0.017)	1.2 × 10^−^^2^	0.016	−0.044 (0.007)	7.4 × 10^−^^11^
6:130,349,119_C/T	*L3MBTL3*	96.46	−0.050 (0.008)	2.6 × 10^−^^9^	−0.038 (0.020)	3.3 × 10^−^^2^	0.010	−0.048 (0.008)	7.8 × 10^−^^10^
7:12,610,578_C/T	*SCIN* (LOF)	3.43	0.055 (0.009)	5.2 × 10^−^^12^	0.040 (0.019)	7.0 × 10^−^^2^	0.011	0.052 (0.008)	1.9 × 10^−^^11^
7:19,529,484_G/C	*FERD3L*, *TWISTNB*	95.01	0.043 (0.007)	2.5 × 10^−^^9^	0.029 (0.017)	1.2 × 10^−^^1^	0.008	0.041 (0.007)	6.2 × 10^−^^10^
7:30,635,521_G/C	*GARS* (EAS)	4.27	0.045 (0.008)	6.0 × 10^−^^9^	0.053 (0.019)	7.1 × 10^−^^3^	0.023	0.047 (0.008)	1.2 × 10^−^^9^
8:19,363,306_G/A	*CSGALNACT1* (NS, EAS)	1.93	−0.085 (0.011)	5.5 × 10^−^^14^	−0.081 (0.026)	1.4 × 10^−^^3^	0.024	−0.084 (0.010)	3.7 × 10^−^^16^
8:57,170,647_A/G	*CHCHD7*, *SDR16C5*	4.60	−0.063 (0.010)	5.0 × 10^−^^9^	−0.097 (0.024)	9.0 × 10^−^^5^	0.082	−0.068 (0.009)	2.5 × 10^−^^13^
9:34,766,707_A/G	*FAM205A*, *FAM205BP* (EAS)	1.09	−0.108 (0.016)	4.8 × 10^−^^12^	−0.154 (0.036)	2.9 × 10^−^^5^	0.057	−0.116 (0.015)	4.0 × 10^−^^15^
9:99,259,568_AGGCGGGGAGGCAGG/A	*HABP4*, *CDC14B*	4.50	0.055 (0.008)	1.0 × 10^−^^13^	0.042 (0.017)	1.9 × 10^−^^2^	0.016	0.053 (0.007)	3.6 × 10^−^^14^
9:109,463,806_C/T	*LINC01505*, *ZNF462* (EAS)	2.14	0.073 (0.012)	2.9 × 10^−^^9^	0.084 (0.027)	5.5 × 10^−^^3^	0.033	0.075 (0.011)	2.6 × 10^−^^11^
11:2,813,322_C/T	*KCNQ1* (R)	1.56	−0.115 (0.015)	7.8 × 10^−^^17^	−0.112 (0.034)	3.5 × 10^−^^3^	0.036	−0.114 (0.013)	1.2 × 10^−^^17^
11:27,016,411_A/G	*FIBIN* (NS, EAS)	3.80	−0.076 (0.009)	1.4 × 10^−^^19^	−0.049 (0.021)	1.6 × 10^−^^2^	0.017	−0.072 (0.008)	4.1 × 10^−^^19^
11:69,253,486_T/C	*MYEOV*, *LINC01488* (EAS)	1.57	−0.068 (0.013)	4.3 × 10^−^^8^	−0.063 (0.031)	6.3 × 10^−^^2^	0.012	−0.067 (0.012)	1.9 × 10^−^^8^
12:14,365,109_T/C	*GRIN2B*, *ATF7IP* (EAS)	1.66	−0.068 (0.012)	1.7 × 10^−^^8^	−0.060 (0.028)	6.4 × 10^−^^2^	0.012	−0.066 (0.011)	2.9 × 10^−^^9^
12:20,668,796_A/G	*PDE3A*	4.75	−0.043 (0.008)	5.8 × 10^−^^10^	−0.024 (0.017)	9.0 × 10^−^^2^	0.005	−0.040 (0.007)	5.1 × 10^−^^9^
12:53,722,361_G/A	*SP7* (NS,EP, EAS)	1.15	−0.164 (0.014)	1.5 × 10^−^^30^	−0.074 (0.035)	2.3 × 10^−^^2^	0.011	−0.151 (0.013)	1.3 × 10^−^^29^
12:66,349,812_A/G	*HMGA2*	4.78	−0.083 (0.007)	2.1 × 10^−^^29^	−0.065 (0.017)	3.8 × 10^−^^4^	0.038	−0.080 (0.007)	1.0 × 10^−^^33^
13:114,301,338_G/A	*TFDP1*, *ATP4B*	3.78	0.053 (0.008)	1.8 × 10^−^^9^	0.035 (0.020)	9.1 × 10^−^^2^	0.008	0.050 (0.008)	1.4 × 10^−^^10^
15:23,8106,52_C/T	*MKRN3*	4.07	−0.068 (0.009)	1.1 × 10^−^^15^	−0.053 (0.021)	1.6 × 10^−^^2^	0.022	−0.066 (0.008)	6.2 × 10^−^^16^
17:42,272,909_C/T	*ATXN7L3*	4.86	−0.047 (0.007)	4.9 × 10^−^^10^	−0.028 (0.016)	8.9 × 10^−^^2^	0.008	−0.044 (0.007)	6.1 × 10^−^^11^
17:54,605,677_G/A	*ANKFN1*, *NOG*	4.28	0.046 (0.009)	1.4 × 10^−^^8^	0.040 (0.020)	2.3 × 10^−^^2^	0.012	0.045 (0.008)	1.3 × 10^−^^8^
19:10,392,379_A/AC	*ICAM1*	3.28	−0.060 (0.010)	1.0 × 10^−^^11^	−0.046 (0.022)	1.8 × 10^−^^2^	0.013	−0.058 (0.009)	5.1 × 10^−^^11^
19:13,264,398_C/T	*IER2* (NS)	2.89	−0.054 (0.009)	5.5 × 10^−^^9^	−0.012 (0.022)	3.3 × 10^−^^1^	0.001	−0.048 (0.009)	2.3 × 10^−^^8^
21:47,549,363_G/C	*COL6A2* (NS, EP, EAS)	1.34	−0.080 (0.016)	4.9 × 10^−^^8^	−0.123 (0.037)	7.2 × 10^−^^4^	0.037	−0.087 (0.015)	2.1 × 10^−^^9^
22:24,087,185_T/C	*ZNF70* (NS)	2.92	−0.059 (0.009)	3.2 × 10^−^^10^	0.007 (0.022)	7.5 × 10^−^^1^	0.000	−0.049 (0.009)	8.3 × 10^−^^9^
X:105,477,754_T/C	*CXorf57* (NS, EAS)	2.70	0.068 (0.010)	4.0 × 10^−^^11^	0.013 (0.026)	6.2 × 10^−^^1^	0.001	0.060 (0.010)	2.4 × 10^−^^10^

Results of the association analysis in GWAS and replication (Rep). Variants with minor allele frequency (MAF) ≥1% and MAF <5% satisfying genome-wide significance after meta-analysis were shown. Annotation: EAS, East Asian specific variant determined by allele frequency in 1000 Genomes Project; NS, variant is in LD with nonsynonymous variant(s); LOF, variant is in LD with loss-of-function variant(s); NL, variant was found in new loci identified by this study; EP, gene is associated with extremely phenotype related to height; R, previously reported variant. Chromosomes and positions are based on Build37 (hg19)

*REF* reference allele, *ALT* alternative allele, *SE* standard error, *VE* variance explained

We searched for 83 coding variants with low MAFs identified in the largest GWAS conducted to date (GIANT consortium^7^) and identified 30 variants evaluated in our GWAS (25 were rare or low-frequency variants in the Japanese population). Among them variants, eight were nominally associated (*P*_GWAS_ <0.05; Supplementary Table 3). We also looked up other height-associated variants in the European population^4–6^ (Supplementary Table 4). Notably, we replicated the association of rs143840904 in the *KCNQ1* region (*P*_GWAS_ = 6.4 × 10^−19^, MAF = 1.6%; Supplementary Fig. 8) that was originally reported by Sardinians^4^. In that previous study, either rs143840904 or rs150199504 was implicated in height based on considering the LD pattern and their allele frequencies in the South Asian population. However, rs150199504 is monomorphic in the BBJ1K and EAS samples of 1KG^9^, and we therefore suspected that rs143840904 was the causal variant of this locus. We also looked up at the associations of variants reported by the meta-analysis of GIANT consortium and UK Biobank (UKB)^8^. By considering that the reported lead variants were selected by GCTA-COJO^17^, we considered 2481 out of 3290 variants showing genome-wide significant associations in the non-conditioned analysis. Of these variants, 1416 variants were evaluated in our GWAS (Supplementary Data 6), and 771 variants were nominally associated (*P*_GWAS_ < 0.05) with the consistent direction of effects. These 771 variants represented a significant positive correlation in the effect sizes between our GWAS and the meta-analysis of GIANT and UKB (Pearson’s *r* = 0.82, *P* = 5.29 × 10^−188^), suggesting that these are shared height-associated variants across populations.

### Functional annotations and pleiotropic effects

We annotated the variants that were in LD (*r*^2^ > 0.8) with the 573 identified variants and found 117 nonsynonymous variants in 98 genes (Supplementary Data 7). Of these genes, 13 have been reported as genes responsible for height-related anomalies in humans (Supplementary Table 5). Among the nonsynonymous variants, 113 were missense variants, 1 was a non-frameshift deletion, and 3 were putative loss-of-function variants, including a low-frequency stop-gain variant (*SCIN* R56X) that is extremely rare in other populations. *SCIN* has been reported to mediate osteoclastogenesis and is involved in developmental dysplasia of the hip^18^.

To characterize rare and low-frequency variants associated with height, we investigated the pleiotropic effects of the identified rare and low-frequency variants across 17 traits by conducting association analysis in the individuals analyzed in GWAS using clinical information obtained from the BBJ (Supplementary Table 6). This analysis revealed three significant pleiotropic associations of two variants (*α* = 4.60 × 10^−5^ (=0.05/(17 × 64)); Supplementary Fig. 9). One was a very rare missense variant in *JAK2* (p.E890K; MAF = 0.08%), which is the causative gene of polycythemia vera (MIM: 263300) and thrombocythemia-3 (MIM: 614521) and is activated by the binding of a growth hormone to a growth hormone receptor. Its height-decreasing allele was associated with lower levels of both red blood cells (*P* for linear regression = 7.90 × 10^−8^, *β* = −0.39, s.e. = 0.07) and hemoglobin (*P* for linear regression = 9.80 × 10^−6^, *β* = −0.32, s.e. = 0.07). The other was a rare missense variant in *IHH* (p.G408R; MAF = 0.30%), which is the known causative gene of acrocapitofemoral dysplasia (MIM: 607778) and brachydactyly type A1 (MIM: 112500). The p.G408R height-decreasing allele also decreased white blood cell levels (*P* for linear regression = 1.02 × 10^−6^, *β* = −0.07, s.e. = 0.01).

### Gene-based analysis

**Fig. 3:**
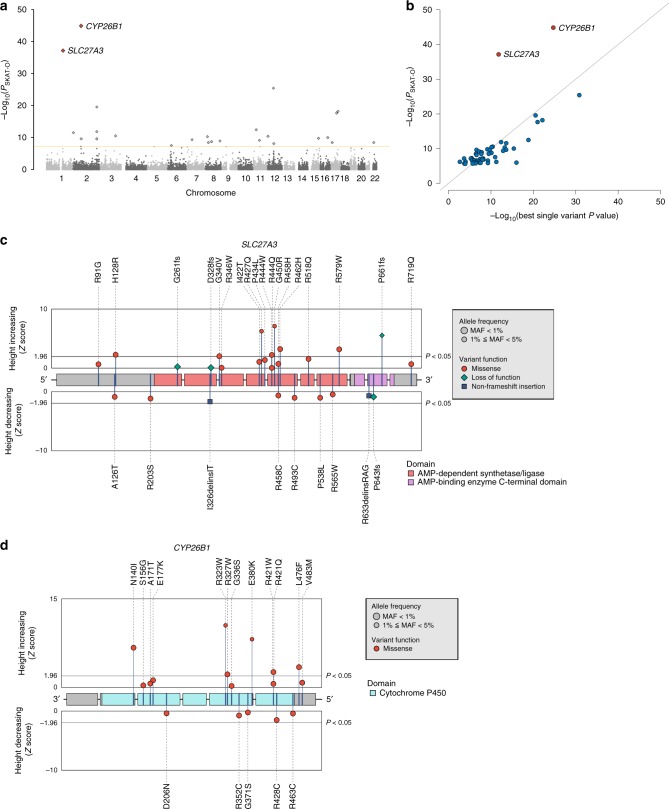
Summary of gene-based analysis. **a** Manhattan plot of the gene-based test. The yellow line represents significance threshold (*P* < 2.5 × 10^−6^). **b** Comparison of single-variant test *P* values and those of the gene-based test. Two genes (*SLC27A3* and *CYP26B1*) showed much stronger associations in the gene-based test. **c**, **d** Positions and associations of nonsynonymous variants in *SLC27A3* and *CYP26B1*. The colors of the plots denote the impact of the variants. The symbols (circle, diamond, and square) indicate the function of variants. The coding regions are colored according to their structural domains based on Pfam

To pinpoint the genes influencing height, we evaluated gene-level associations with SKAT-O^19^ in samples analyzed through GWAS and identified 52 genes that were significantly associated with height (*P*_SKAT-O_ < 2.5 × 10^−6^; Fig. 3a and Supplementary Data 8). Although most of the observed gene-level associations seemed to represent the single best-associated nonsynonymous variants in each gene, we found stronger associations by grouping other nonsynonymous variants in the two genes *SLC27A3* (*P*_SKAT-O_ = 6.94 × 10^−38^) and *CYP26B1* (*P*_SKAT-O_ = 1.33 × 10^−45^) (Table 3 and Fig. 3b), indicating multiple associated low-frequency variants (Fig. 3c, d). All of the minor alleles of the variants showing a nominal association (*P*_GWAS_ < 0.05) exerted height-increasing effects in both genes (Supplementary Data 9, 10). The replication sets confirmed the associations (Table 3). To further investigate whether the identified gene-level associations of two genes resulted from the associations of multiple associated variants, we performed a conditional analysis. We observed significant gene-level associations remained after conditioned on the best-associated single nonsynonymous variant (*P*_SKAT-O_ = 3.38 × 10^−25^, and 2.41 × 10^−21^ for *SLC27A3* and *CYP26B1*, respectively; Supplementary Table 7). The missense variant in *CYP26B1* (p.L264S) was found to show a subgenome-wide significant association in an African population in the recent analysis by the GIANT consortium (*P* = 1.13 × 10^−7^)^7^. We confirmed that this variant showed an association in both our GWAS (*P*_GWAS_ = 2.5 × 10^−7^, MAF = 9.2%) and the replication set (*P*_rep_ = 4.8 × 10^−3^, MAF = 9.4%) (Supplementary Data 10). Although we did not include *CYP26B1* p.L264S in the gene-based analysis, due to its higher MAF, this observation also supported the conclusion that multiple nonsynonymous variants of *CYP26B1* are associated with human height.

**Table 3:** Gene-level associations of *SLC27A3* and *CYP26B1*

Gene	Chr:Pos	*N* variants in GWAS^a^	*P*_GWAS_^b^	*N* variants in replication^a^	*P*_rep_^b^
*SLC27A3*	1:153,748,103–153,752,478	27	6.94 × 10^−38^	27	7.03 × 10^−5^
*CYP26B1*	2:72,359,400–72,374,900	17	1.33 × 10^−45^	18	3.91 × 10^−7^

Chromosomes and positions are based on Build37 (hg19)

^a^A number of variants were used in gene-based analysis. Variants with an imputation quality score of Rsq ≥0.3 were employed in the analysis

^b^*P* values were calculated by SKAT-O

Disruption of *CYP26B1* gene has been reported to cause fusion or overgrowth of cartilaginous structures in mice and zebrafish and leads to craniosynostosis and multiple skeletal anomalies in humans^20^; however, the impact of *SLC27A3* on human phenotypes has not been well characterized. Therefore, we evaluated their pleiotropic effects on 17 human traits and found that *SLC27A3* showed significant associations with lipid-related traits (*P*_SKAT-O_ = 4.30 × 10^−4^ and 8.20 × 10^−5^, for total cholesterol and triglyceride, respectively), and *CYP26B1* influenced the body-mass index (*P*_SKAT-O_ = 2.03 × 10^−4^) (Supplementary Fig. 10). Considering that *SLC27A3* is the fatty acid transporter (*FATP*) gene, it is likely that further functional investigation of *SLC27A3* will reveal the biological link of *FATP* genes with the regulation of height.

### Pathway analysis

To gain further biological insight, we performed a gene set enrichment analysis using PASCAL^21^ with reconstituted gene sets, which was implemented in DEPICT^22^. We observed significant enrichment in 221 gene sets (false discovery rate (FDR) *q* < 0.05; Supplementary Data 11). Of these 221 significant gene sets, 37 gene sets were not reported in the previous study of GIANT consortium^7^. Since these gene sets were correlated with each other, we clustered the identified gene sets into 16 categories using the affinity propagation (AP) clustering method^23^ and determined the exemplars in each category. Although gene sets represented by regulatory region DNA binding (GO: 00010607) have not been previously reported, the other 15 categories contained one or more gene sets previously reported by the GIANT consortium^7^, and the overall observations should support the notion that the biological mechanisms regulating human height are shared across different populations.

### Variance explained by the analyzed variants

Next, we estimated the phenotypic variance explained by the variants analyzed in the replication sets (*N* = 25,419) using the MAF-stratified genomic-relatedness-based restricted maximum likelihood (GREML-MS) method^24^ with GCTA^25^, which enabled us to evaluate the explained variance of the sets of variants stratified by MAF. When we investigated all imputed autosomal variants, it was estimated that 52.4 ± 4.5% of the total phenotypic variance could be explained. Variants with a MAF <10% explained 20.3 ± 4.6% of the variance. We further split the low-frequency range at a MAF of 5% and found that variants with a MAF <5% explained the largest proportion of the phenotypic variance in human height (14.1 ± 4.7%; Supplementary Fig. 11). Considering that the identified rare and low-frequency variants explained only 1.7% of the phenotypic variance in our samples, further investigations of rare and low-frequency variants are warranted to elucidate the genetic architecture of human height.

### Analysis of natural selection

**Fig. 4:**
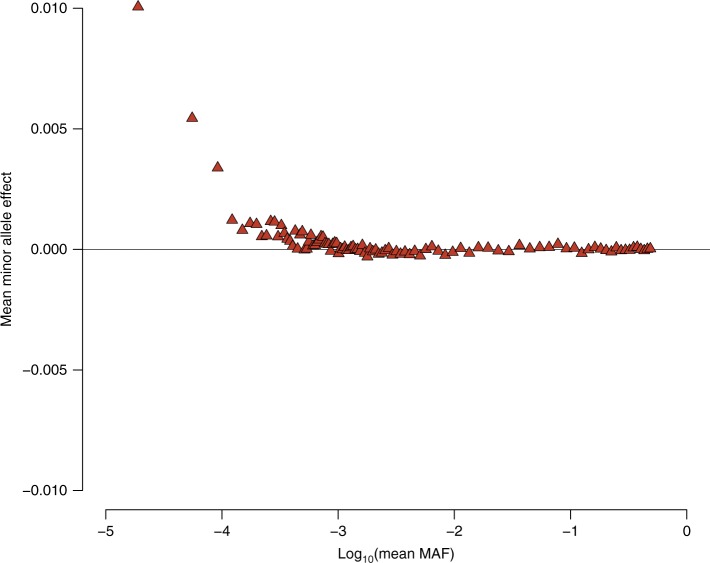
Relationship between the minor allele effect for height and MAF in the GWAS. A scatter plot of the mean minor allele effect according to each MAF bin (*N* = 100) is shown. The mean effects of the minor alleles were clearly greater in rarer variants

Recent studies have indicated that the variants associated with human height are under natural selection^4,24,26^; however, such evidence has been limited to the European population. By examining the relationships between allele frequencies and effect sizes through GWAS, we investigated the effect of selection on height-associated variants in the Japanese population (Fig. 4). We observed that the rarer variants tend to have height raising effects (Spearman’s *ρ* = −0.63, *P* < 1.0 × 10^−6^), implicating negative selection on height-increasing alleles. This relationship showed a clear contrast to findings in Europeans (in whom rarer variants entirely showed height-decreasing tendency after grouped by their MAF)^24^. The independent replication set confirmed this finding (Spearman’s *ρ* = −0.69, *P* < 1.0 × 10^−6^, Supplementary Fig. 12). This dissimilarity implies differences in selection pressure on height-associated variants in the Japanese and European populations.

Finally, we assessed the possible artifacts in the analysis of relationship between minor allelic effects (MAEs) and MAFs. First, we considered the bias resulting from genotype imputation. We evaluated this tendency using the directly genotyped variants, and observed the consistent relationship between MAEs and MAFs (Supplementary Fig. 13; Spearman’s *ρ* = −0.39, *P* = 7.38 × 10^−6^), suggesting that the observed height-increasing tendency in rarer variants did not result from the bias emerged from imputation. We also considered the impact of convergence in the BOLT-LMM on this analysis. We therefore restricted the variants with standard errors of estimates ≤0.2 and 0.5, and re-evaluated the relationship between MAFs and MAEs. We observed similar relationship (Supplementary Fig. 14; Spearman’s *ρ* = 0.61 and 0.69 for variants with SE < 0.2 and 0.5, respectively). Based on these results, we interpreted that the impact of convergence on our result is minimal. Next, in order to assess the possible contribution of population stratification, we selected the samples belonging to the Japanese main cluster and performed GWAS (Supplementary Fig. 15a; *N* = 148,937). When we evaluated the correlation between MAFs and MAEs, we still observed significant correlation (Supplementary Fig. 15b; Spearman’s *ρ* = −0.35, *P* = 3.35 × 10^−4^).

## Discussion

Through a large-scale genome-wide analysis in a single non-European population, we identified 64 rare and low-frequency variants and two genes associated with human height. The discovery of the rare and low-frequency height-associated variants was led by the integrated reference panel for genotype imputation, which was constructed with population-specific whole-genome sequencing data^10^. Considering that dozens of the identified genetic variants appeared to be specific to East Asians, investigations of rare and low-frequency variants in non-European populations will contribute to expand the catalog of genes and genetic variations associated with complex traits. Our findings should encourage further investigations of diverse populations in complex trait genetics.

Our results suggested similarities of genetic architecture of human height between Europeans and East Asians. First, we showed the correlation of effect sizes of height-associated variants between East Asians and Europeans, implying that a certain proportion of height-associated variants are shared across populations. Second, the pathway analysis implicated that the large proportion of the identified gene sets were shared between ours and those found in Europeans. This observation supports the shared biological mechanisms and genetic contributions on human height. Third, the estimated heritability stratified by MAF was comparable between ours and the previous study of Europeans. These observations will help to deepen our knowledge on genetic architecture of human height. On the other hand, we showed some advantages of investigating non-European population, such as identification of dozens of the East Asian-specific height-associated variants, the likely causal variant at *KCNQ1* region, and two novel gene-level associations, which were not reported in the previous study. Moreover, by utilizing the phenotypic resources of the Biobank in Japanese population, we evaluated the pleiotropy of the identified rare and low-frequency variants and genes, and found three pleiotropic effects of two rare variants and three gene-level pleiotropic associations in two newly identified genes with multiple rare and low-frequency height-associated variants. These findings contribute to characterize the rare and low-frequency variants associated with height in the Japanese population.

We investigated the negative selection on height-associated variants, and found the relationship that rarer variants showed height-increasing tendency. We note that this tendency appeared for the grouped variants with mean MAF of at most 0.1%. Among the lead variants significantly associated in the present study, only one variant (rs202237966) was MAF <0.1%, suggesting that investigation of the negative selection using significantly associated variants requires much larger sample size. Considering that investigation of rare variants in a Japanese population suggested difference in negative selection on human height, further multi-ethnic comparisons of rare genetic components may expand our knowledge on the selection pressures underlying complex traits. On the other hand, the correlation in the Japanese main cluster was weakened, suggesting the possible bias from population stratification. Future study using novel statistical method, which is not influenced by population stratification, will be needed to confirm our finding.

In conclusion, we performed a large-scale genetic association study in the Japanese population, and identified 573 variants including 22 rare and 42 low-frequency variants associated with height in the Japanese population. Our results provide insights into the similarities and differences underlying genetic architecture of human height, and underscore the utility of investigating diverse populations to deepen our understanding of the genetic architecture of human complex traits.

## Methods

### Subjects

Clinical information, including height, and the genome-wide variant data of the samples included in the GWAS were obtained from the BBJ project^11,12^, which enrolled 200,000 participants. We set the eligibility criteria for the GWAS samples as follows: (1) age ≥ 18, (2) available height information, and (3) height within three times the interquartile range of the upper/lower quartile. We calculated *Z*-scores from the residuals of linear regression against height using age, age^2^, and sex, and excluded individuals who fell outside ±4 standard deviations. We used genotyping array results to further exclude samples with lower call rates (<98%), closely related individuals using PLINK^27^ (PI_HAT > 0.175). We determined outliers from the Japanese cluster by visual inspection based on the principal component analysis (PCA) using GCTA software^25^ (ver1.25) with the samples of HapMap project^28^ (Supplementary Fig. 16). Finally, 159,095 individuals were included in the GWAS. There was no overlap between samples included in the GWAS and BBJ1K.

The samples used in the replication study were collected from four Japanese population-based studies (Iwate Tohoku Megabank Organization (IMM), Japan Multi-Institutional Collaborative Cohort Study (JMICC), Japan Public Health Center-based Prospective Study (JPHC), and Tohoku University Tohoku Medical Megabank Organization (ToMMo)). We applied the same inclusion and exclusion criteria to the clinical information of the participants. To increase statistical power, we excluded overlapping samples (estimated by PI_HAT) and PCA outliers from the East Asian population and finally included 32,692 individuals in the replication study.

In all participating studies, informed consent was obtained from all participants by following the protocols approved by the corresponding institutional ethical committees before enrollment. The clinical characteristics of each cohort are provided in the Supplementary Table 1. This study was approved by the ethics committee of RIKEN and the Institute of Medical Science, the University of Tokyo.

### Imputation reference panel construction

To achieve better imputation accuracy, we constructed a reference panel using WGS data obtained from the BBJ^10^. Briefly, we sequenced 1037 samples using 2 × 160-bp paired-end reads on the HiSeq2500 platform, aimed at a 30× depth, and processed these data according to the standardized best-practice method proposed by GATK (ver.3.2-2). For QC, we additionally set exclusion filters for genotypes as follows: (1) DP <5, (2) GQ <20, or (3) DP >60 and GQ <95. We set these genotypes as missing and excluded variants with call rates <90% before variant quality score recalibration (VQSR). After performing VQSR implemented by GATK, variants located in low-complexity regions (LCR), as defined by mdust software (“hs37d5-LCRs.20140224.bed,” downloaded from ftp://ftp.1000genomes.ebi.ac.uk/vol1/ftp/release/20130502/supporting/low_complexity_regions/hs37d5-LCRs.20140224.bed.gz), were excluded. Finally, we used BEAGLE to impute missing genotypes.

To combine the 1KG phase3v5 (*n* = 2504) and BBJ1K data using IMPUTE2^29,30^, we excluded variants located at multi-allelic sites and estimated haplotypes using SHAPEIT. After combining the two datasets, we excluded singletons and variants at multi-allelic sites from the reference panel using bcftools (ver. 1.3.1) and vcftools (ver. 0.1.14). Since the current version of IMPUTE2 is not applicable to haploid chromosomes, we used BEAGLE to impute WGS data for X chromosomes in males and excluded singletons and variants located at multi-allelic sites before merging with a female dataset.

To evaluate the imputation accuracy of different reference panels, we performed whole-chromosome 1 imputation in GWAS samples after excluding variants that were included on the Illumina HumanExome BeadChip. For the imputation reference panels, we used the genotype data from the following samples: (1) 1KG phase1v3 EAS samples, (2) 1KG phase3v5 ALL samples, (3) BBJ1K samples, and (4) the combined 1KG phase3v5 ALL and BBJ1K samples. We carried out pre-phasing and imputation using Eagle (v2)^31^ and minimac3^32^, respectively. The accuracy of the imputation was evaluated by Pearson’s *r*^2^ between the GWAS array genotypes and the imputed allelic dosages.

### Genotyping and imputation

Samples included in the GWAS were genotyped using either of the following genotyping arrays: (1) the Illumina HumanOmniExpressExome BeadChip or (2) a combination of the Illumina HumanOmniExpress and HumanExome BeadChips. For QC of genotypes, we excluded variants meeting any of the following criteria: (1) call rate <99%, (2) *P* value for Hardy–Weinberg equilibrium <1.0 × 10^−6^, and (3) number of heterozygotes <5. After we proceeded through these QC steps using all genotyped samples in the BBJ set, 939 samples from BBJ1K were included. We further compared the genotypes of the overlapping variants between the GWAS array and WGS and excluded variants with a concordance rate <99.5% or a non-reference concordance rate ≥0.5%. In the GWAS, we used Eagle^31^ for haplotype phasing without an external reference panel and Minimac3^32^ for imputation, respectively. For the purpose of QC, we used variants with an Rsq ≥0.3 in the association analysis.

In the replication set, all participants were genotyped with the Illumina HumanOmniExpressExome BeadChip. We excluded variants meeting any of the following criteria: (1) call rate <98%, (2) *P* value for Hardy–Weinberg equilibrium <1.0 × 10^−6^, and (3) a minor allele count of <5. Thereafter, we compared the allele frequency between the genotyped samples and BBJ1K and excluded variants showing a >5% difference in allele frequency. Pre-phasing was carried out using SHAPEITv2^33^. We imputed variants with Minimac3 using the same reference panel as in the GWAS. We considered variants with an Rsq ≥0.3 in the association analysis.

Pre-phasing and imputation of the X chromosome were performed using the same software applied for autosomes. In each dataset, we phased the haplotypes for males and females together and then imputed them separately in each sex.

### Phenotype and association analysis

For the single-variant association analysis, we obtained the residuals from a linear regression model of height adjusted for age, age^2^, and sex. The residuals were then converted into *Z*-scores. Association analysis of autosomes was performed using BOLT-LMM (ver.2.2), which implements linear mixed model analysis under the Gaussian mixture model^13^. Since this approach is not applicable to the X chromosome, we recalculated the residuals of the linear regression analysis by adding the top 10 principal components (PCs) of the genotypes as covariates and converted them into *Z*-scores. Association analysis of the X chromosome was carried out with mach2qtl^32^ in the GWAS and with EPACTS (ver. 3.2.6) in the replication study. Since we did not remove related samples from the replication set, we additionally excluded samples based on a PI_HAT >0.175 and included 30,303 samples in the X-chromosomal analysis.

In the conditional analysis performed in the GWAS, we used BOLT-LMM for autosomes and mac2qtl for the X chromosome. We set the same significance threshold employed for the GWAS (*α* = 5.0 × 10^−8^) in the conditional analysis and repeated the analysis until the association of the top variants fell below the significance threshold. To replicate the associations of variants identified through conditional analysis, we used the same set of conditioned variants employed in the GWAS as covariates in the replication set.

We integrated the results of the GWAS and the replication set using the inverse-variance method and estimated heterogeneity with Cochran’s *Q* test. The variants that satisfied genome-wide significance (*P* < 5.0 × 10^−8^) after the meta-analysis were considered to be significantly associated throughout the study.

For the evaluation of gene-level associations, we conducted a group-wise association analysis of coding regions in autosomal genes. For phenotype preparation, we obtained the residuals of the linear regression of height with age, age^2^, sex, and the top 10 PCs as covariates and standardized them as *Z*-scores. We annotated the variants as described below and extracted nonsynonymous variants, including missense and nonsense variants, non-frameshift insertions and deletions, and frameshift variants with a MAF <5% and imputation quality of Rsq ≥0.3. A group-wise association test was performed using the imputed allelic dosage employing SKAT-O^19^ implemented in EPACTS. As in the X-chromosome analysis, we excluded closely related samples from the replication set. We set a conservative significance threshold for the gene-level association analysis using Bonferroni correction based on the assumption that 20,000 genes could be evaluated (*α* = 0.05/20,000 genes). To evaluate whether the identified gene-level associations resulted from multiple nonsynonymous variants, we performed conditional analyses by adjusting for the best-associated variant of each gene included in the group-wise association analysis.

### LD score regression

To assess bias resulted from population stratification and cryptic relatedness, we conducted a single variate LD score regression^14^ analysis using LDSC v.1.0.0. We used the LD score file for East Asian population provided with the software.

### Annotation

We annotated variants using ANNOVAR^34^ (version: 2017-07-17). PLINK^27^ was employed to estimate LD with the lead variant of each identified locus in the BBJ1K samples. Protein domain information for *SLC27A3* and *CYP26B1* from Pfam^35^ was obtained via the Ensembl genome browser.

### Pleiotropy

To investigate the pleiotropic effects of the 64 identified variants with a MAF <5%, we evaluated the associations of 17 different phenotypes using the clinical information of the samples analyzed in the GWAS. We targeted the nine quantitative traits among hematological, kidney-related and liver-related traits and two diseases by considering the results of our genetic correlation analysis across 89 complex traits^16^. We additionally selected four lipid-related traits, hemoglobin A1c, and type 2 diabetes because height-associated rare and low-frequency variants showed significant associations with these traits in a previous study^7^. For quantitative traits, we used standardized phenotypes (detailed in Supplementary Table 6) and performed linear regression analysis. For the case–control analysis, we applied a logistic regression analysis adjusted for age, sex, and the top 10 PCs as covariates.

We also tested the pleiotropy of the identified genes (*SLC27A3* and *CYP26B1*) for the same 17 traits. Gene-level associations were estimated with SKAT-O^19^ using EPACTS (ver. 3.2.6), with the same settings applied for height. We set the significance threshold using Bonferroni correction at *α* = 0.05/17/64 for single-variant analysis and *α* = 0.05/17/2 for the gene-based test.

### Pathway analysis

We conducted a gene set enrichment analysis using PASCAL software^21^. To fit into the Japanese LD structure, we used a custom reference panel generated from the BBJ1K set. We used 14,462 gene sets reconstituted with DEPICT software^22^ (*Z*-score >3; software downloaded from https://data.broadinstitute.org/mpg/depict/depict_download/reconstituted_genesets/).

The default settings were employed for the analysis. After determining significant gene sets by evaluating the FDR using the Benjamini–Hochberg method (threshold: FDR <0.05) for the *P* value estimated via the *χ*^2^ pathway scoring method, we applied an AP clustering method^23^ to merge the identified gene sets into functionally relevant categories and defined exemplars in each category. We used the *Z*-scores for all genes to perform this clustering.

### GREML analysis

We employed Japanese population-based cohorts (replication set) to estimate the variance explained by the imputed variants. Following a previous report^24^, we used hard call genotypes (*x*_dose_) estimated based on the imputed allelic dosage (*x*_g_), without excluding variants with a lower imputation quality. Here, we converted *x*_g_ = 0 for *x*_dose_ < 0.5; *x*_g_ = 2 for *x*_dose_ > 1.5; and *x*_g_ = 1 for 0.5 ≤ *x*_dose_ ≤ 1.5; and we excluded variants with a minor allele count <3 and a *P* for Hardy–Weinberg <1.0 × 10^−6^. To estimate the variance explained by the variants contained in our reference panel, the GREML-MS^24^ approach was applied using GCTA software^25^ (ver. 1.25). We first generated a genetic relationship matrix (GRM) using all of the variants and estimated genetic relatedness and restricted samples with relatedness <0.05 using the “–grm-cutof’” option. As a result, 25,419 samples were included in the analysis. Trait values were re-standardized in these samples. Subsequently, we generated seven GRMs stratified by MAF as follows: (1) MAF <5%, (2) 5% ≤ MAF < 10%, (3) MAF <10%, (4) 10% ≤ MAF < 20%, (5) 20% ≤ MAF < 30%, (6) 30% ≤ MAF < 40%, and (7) 40% ≤ MAF. We estimated phenotypic variance according to each MAF strata using the “–reml” option with the top 10 PCs to control population stratification.

### Mean allelic effect stratified by MAF

We evaluated the relationship between the MAF and the minor allele effect in our samples by utilizing the variants analyzed in the GWAS (Rsq ≥0.3). After excluding the variants that did not converge, we divided the autosomal variants into 100 bins according to their MAF. The number of the variants in each bin was determined as follows. First, we divided the number of all the analyzed variants by 100. The number of variants assigned in bins 1–99 was the integer quotient of the number of all the variants +1, and the remained variants were grouped into the bin 100. Then, we estimated Spearman’s rank correlation coefficient (*ρ*) between the MAF and the minor allele effect in each bin. The statistical significance of a correlation coefficient value was determined via one million resamplings. In each resampling procedure, we randomly selected the same number of variants and estimated the empirical distribution of *ρ* values, as proposed previously^24^. For the purpose of replication, we analyzed samples in the replication set in the same way.

### URLs

For BBJ, see https://biobankjp.org/english/index.html; for IMM, see http://iwate-megabank.org/en/; for JMICC, see http://www.jmicc.com/en/; for JPHC, see http://epi.ncc.go.jp/en/jphc/index.html; for ToMMo, see http://www.megabank.tohoku.ac.jp/english/; for PLINK, see https://www.cog-genomics.org/plink2; for BOLT-LMM, see https://data.broadinstitute.org/alkesgroup/BOLT-LMM/downloads/; for MACH, see http://csg.sph.umich.edu//abecasis/MaCH/; for ANNOVAR, see http://annovar.openbioinformatics.org/en/latest/; for SHAPEIT, see https://mathgen.stats.ox.ac.uk/genetics_software/shapeit/shapeit.html; for Eagle, see https://data.broadinstitute.org/alkesgroup/Eagle/; for R, see https://www.r-project.org/; for GIANT consortium, see https://www.broadinstitute.org/collaboration/giant/index.php/GIANT_consortium; for Locuszoom, see http://locuszoom.sph.umich.edu/locuszoom/; for 1000 genome project, see http://www.1000genomes.org/; for GCTA, see http://cnsgenomics.com/software/gcta/; for PASCAL, see https://www2.unil.ch/cbg/index.php?title = Pascal; for EPACTS, see https://genome.sph.umich.edu/wiki/EPACTS; for IMPUTE2, see https://mathgen.stats.ox.ac.uk/impute/impute_v2.html; for GATK, see https://software.broadinstitute.org/gatk/; for Beagle, see https://faculty.washington.edu/browning/beagle/beagle.html

### Reporting summary

Further information on research design is available in the Nature Research Reporting Summary linked to this article.

## Supplementary information

Supplementary Information

Reporting Summary

Description of Additional Supplementary Files

Supplementary Data 1

Supplementary Data 2

Supplementary Data 3

Supplementary Data 4

Supplementary Data 5

Supplementary Data 7

Supplementary Data 6

Supplementary Data 8

Supplementary Data 9

Supplementary Data 10

Supplementary Data 11

## Data Availability

The summary statistics for the GWAS are available from the National Bioscience Database Center (NBDC) Human Database (https://humandbs.biosciencedbc.jp/en/) with the dataset ID (hum0014 [https://humandbs.biosciencedbc.jp/en/hum0014-v15]) and from JENGER (http://jenger.riken.jp/en/). The genotype data employed in the GWAS are available from the Japanese Genotype-phenotype Archive (JGA; http://trace.ddbj.nig.ac.jp/jga/index_e.html) with accession codes JGAS00000000114 for the study and JGAD00000000123 for the genotype data. The constructed reference panel for genotype imputation is also accessible through JGA upon request (accession code: JGAD00000000220). Height information can be provided by the BBJ project upon a request (please see, https://biobankjp.org/english/index.html).
