# Author Correction: Characterizing rare and low-frequency height-associated variants in the Japanese population

**DOI:** 10.1038/s41467-020-15202-2

**Published:** 2020-03-09

**Authors:** Masato Akiyama, Kazuyoshi Ishigaki, Saori Sakaue, Yukihide Momozawa, Momoko Horikoshi, Makoto Hirata, Koichi Matsuda, Shiro Ikegawa, Atsushi Takahashi, Masahiro Kanai, Sadao Suzuki, Daisuke Matsui, Mariko Naito, Taiki Yamaji, Motoki Iwasaki, Norie Sawada, Kozo Tanno, Makoto Sasaki, Atsushi Hozawa, Naoko Minegishi, Kenji Wakai, Shoichiro Tsugane, Atsushi Shimizu, Masayuki Yamamoto, Yukinori Okada, Yoshinori Murakami, Michiaki Kubo, Yoichiro Kamatani

**Affiliations:** 1 Laboratory for Statistical Analysis, RIKEN Center for Integrative Medical Sciences, Yokohama, 230-0045 Japan; 2 Department of Ophthalmology, Graduate School of Medical Sciences, Kyushu University, Fukuoka, 812-8582 Japan; 3 Laboratory for Statistical and Translational Genetics, RIKEN Center for Integrative Medical Sciences, Yokohama, 230-0045 Japan; 4 Department of Allergy and Rheumatology, Graduate School of Medicine, The University of Tokyo, Tokyo, 113-8655 Japan; 5 Laboratory for Genotyping Development, RIKEN Center for Integrative Medical Sciences, Yokohama, 230-0045 Japan; 6 Laboratory for Endocrinology, Metabolism and Kidney Diseases, RIKEN Center for Integrative Medical Sciences, Yokohama, 230-0045 Japan; 7 Laboratory of Genome Technology, Human Genome Center, Institute of Medical Science, The University of Tokyo, Tokyo, 108-8639 Japan; 8 Laboratory of Clinical Genome Sequencing, Department of Computational Biology and Medical Sciences, Graduate school of Frontier Sciences, The University of Tokyo, Tokyo, 108-8639 Japan; 9 Laboratory for Bone and Joint Diseases, RIKEN Center for Integrative Medical Sciences, Tokyo, 108-8639 Japan; 10 Department of Genomic Medicine, Research Institute, National Cerebral and Cardiovascular Center, Osaka, 565-8565 Japan; 11 Department of Biomedical Informatics, Harvard Medical School, Boston, MA02115 USA; 12 Nagoya City University Graduate School of Medical Sciences, Nagoya, 467-8601 Japan; 13 Department of Epidemiology for Community Health and Medicine, Kyoto Prefectural University of Medicine, Graduate School of Medical Science, Kyoto, 602-8566 Japan; 14 Department of Preventive Medicine, Nagoya University Graduate School of Medicine, Nagoya, 466-8550 Japan; 15 Department of Oral Epidemiology, Graduate School of Biomedical and Health Sciences, Hiroshima University, Hiroshima, 734-8553 Japan; 16 Division of Epidemiology, Center for Public Health Sciences, National Cancer Center, Tokyo, 104-0045 Japan; 17 Iwate Tohoku Medical Megabank Organization, Iwate Medical University, Iwate, 028-3694 Japan; 18 Department of Hygiene and Preventive Medicine, School of Medicine, Iwate Medical University, Iwate, 028-3694 Japan; 19 Tohoku Medical Megabank Organization, Tohoku University, Sendai, 980-8573 Japan; 20 Graduate School of Medicine, Tohoku University, Sendai, 980-8575 Japan; 21 Center for Public Health Sciences, National Cancer Center, Tokyo, 104-0045 Japan; 22 Department of Statistical Genetics, Osaka University Graduate School of Medicine, Osaka, 565-0871 Japan; 23 Laboratory of Statistical Immunology, Immunology Frontier Research Center (WPI-IFReC), Osaka University, Osaka, 565-0871 Japan; 24 Division of Molecular Pathology, The Institute of Medical Science, The University of Tokyo, Tokyo, 108-8639 Japan; 25 RIKEN Center for Integrative Medical Sciences, Yokohama, 230-0045 Japan; 26 Laboratory of Complex Trait Genomics, Department of Computational Biology and Medical Sciences, Graduate School of Frontier Sciences, The University of Tokyo, Tokyo, 108-8639 Japan

**Keywords:** Genome-wide association studies, Heritable quantitative trait, Rare variants, Quantitative trait

Correction to: *Nature Communications* 10.1038/s41467-019-12276-5, published online 27 September 2019.

In the original version of this paper, under the subheading “Genome-wide association study” in the last-but-one sentence, the number of variants included in the analysis of overlap between GWAS variants in the BBJ and in European population GWAS was incorrectly given as 1416, leading to incorrect association results. The correct number of analysed variants is 1417 and the sentences should read “Of these variants, 1417 variants were evaluated in our GWAS (Supplementary Data 6), and 772 variants were nominally associated (*P*_GWAS_ < 0.05) with the consistent direction of effects. These 772 variants represented a significant positive correlation in the effect sizes between our GWAS and the meta-analysis of GIANT and UKB (Pearson’s *r* = 0.82, *P* = 7.00 × 10^−189^), suggesting that these are shared height-associated variants across populations.”

In addition, the list of variants in Supplementary Data 6 (“Associations of Japanese GWAS at reported variants in large-scale European GWAS”) was incorrect. Two out of 1417 evaluated variants were inadvertently omitted from the table while 460 variants were included by mistake (based on overlap of BBJ GWAS with 3290 lead variants from European GWAS instead of 2481 variants from the nonconditioned analysis, which would have been correct). The corrected Supplementary Data 6 lists the correct 1417 variants obtained in this analysis.

Finally, there was a typo in the methods section, under the subheading “Genotyping and imputation” in the fourth sentence. The sentence originally read “We further compared the genotypes of the overlapping variants between the GWAS array and WGS and excluded variants with a concordance rate <99.5% or a non-reference concordance rate ≥0.5%” where the second “concordance” (in “non-reference concordance rate ≥ 0.5%”) should have been “discordance”.

All of these errors have now been corrected in the HTML and PDF versions of the article and the Supplementary Data 6 file has been updated in the HTML version of the article.

