# Supplementary material for: Characterizing rare and low-frequency height-associated variants in the Japanese population
Source: Nat Commun. 2019 Sep 27;10:4393. doi: 10.1038/s41467-019-12276-5 (PMC6764965; doi:10.1038/s41467-019-12276-5)
Supplement: Supplementary file 1 — Supplementary Information [file 41467_2019_12276_MOESM1_ESM.pdf]

Supplementary Information

Characterizing rare and low-frequency height-associated variants in the Japanese population

Akiyama M et al.

Table of Contents:

Supplementary Figures………………………………………………… 2

Supplementary Tables…………………………………………………. 19

Supplementary Notes………………………………………………….. 28

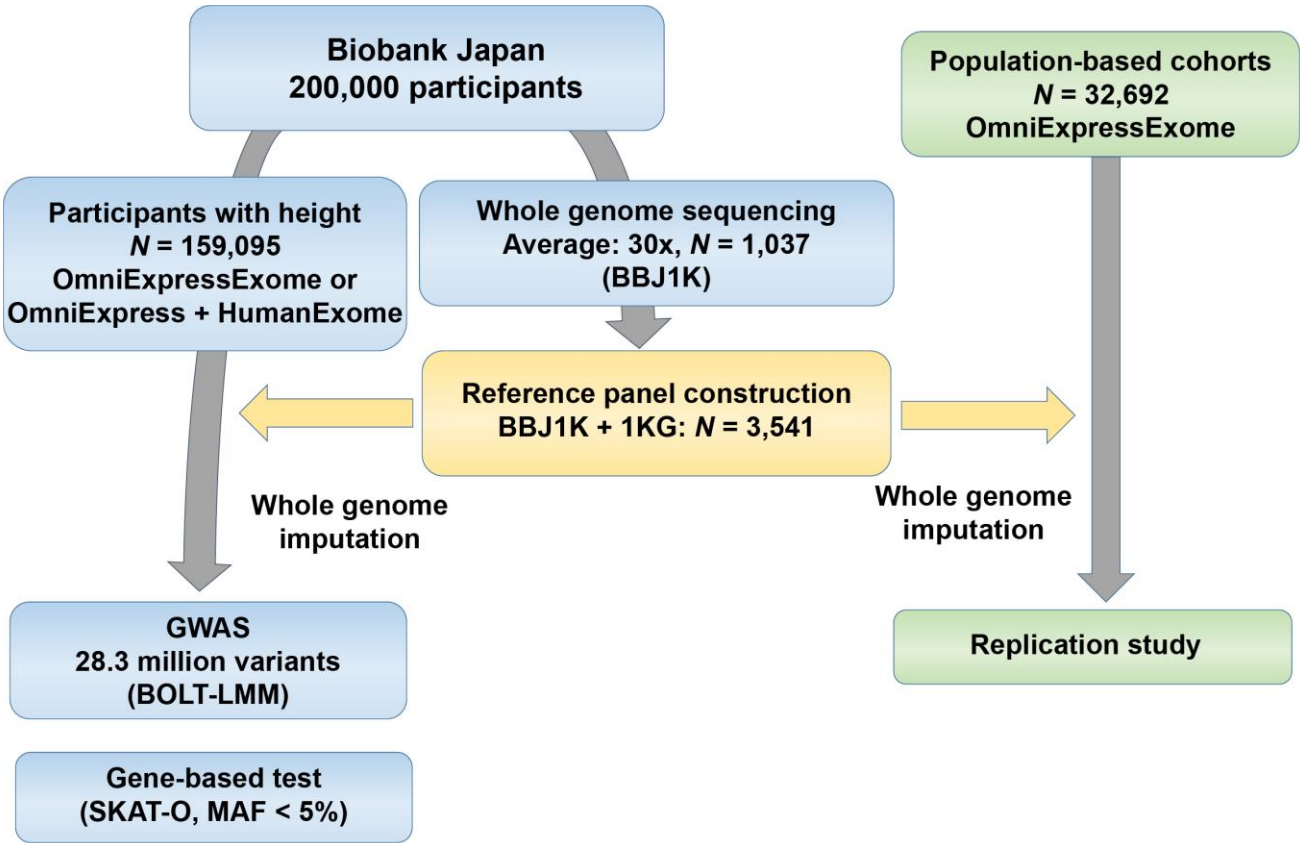

Supplementary Figure 1 | Overview of the study

The overview of the study is illustrated.

a

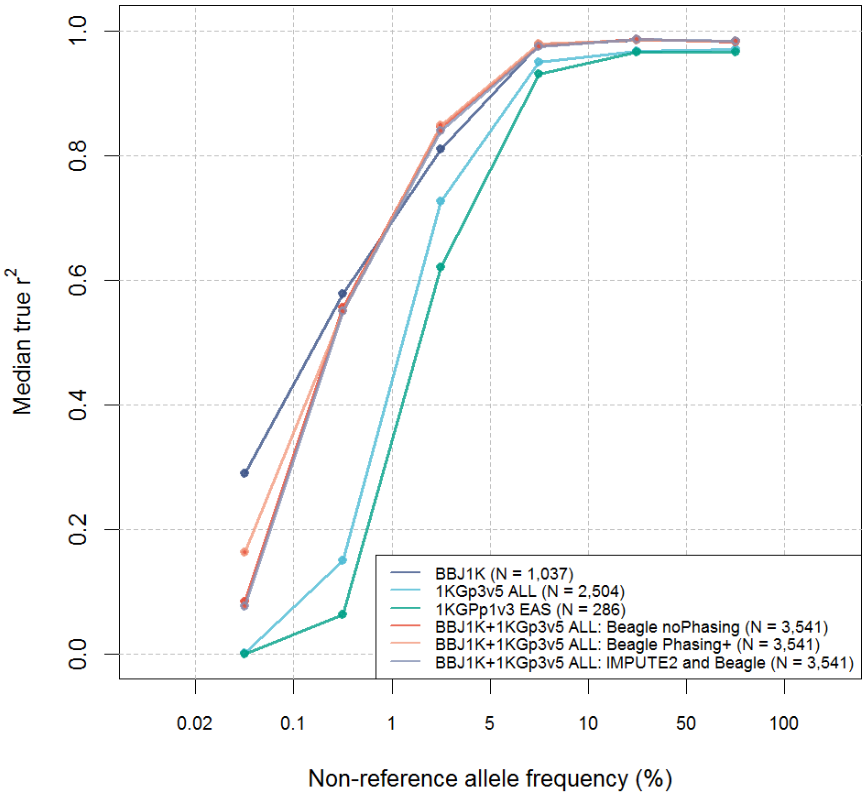

b

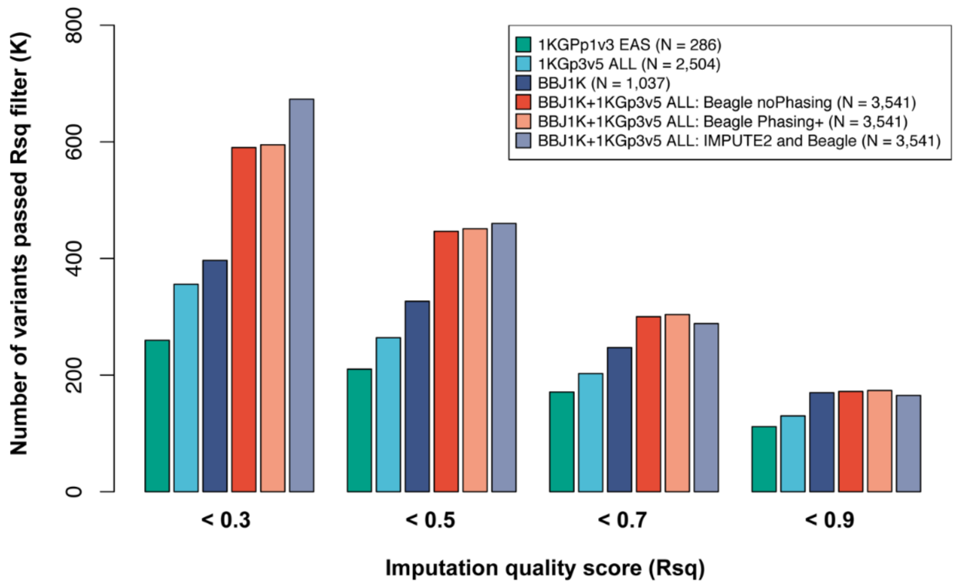

Supplementary Figure 2 | Evaluation of imputation qualities of X-chromosomal variants by different reference panels

The accuracy of imputation was evaluated for six different reference panels at “masked” HumanExome SNPs in X-chromosome (a). We counted the number of variants satisfied the imputation quality scores (Rsq < 0.3, 0.5, 0.7, and 0.9) in each dataset (b).

a

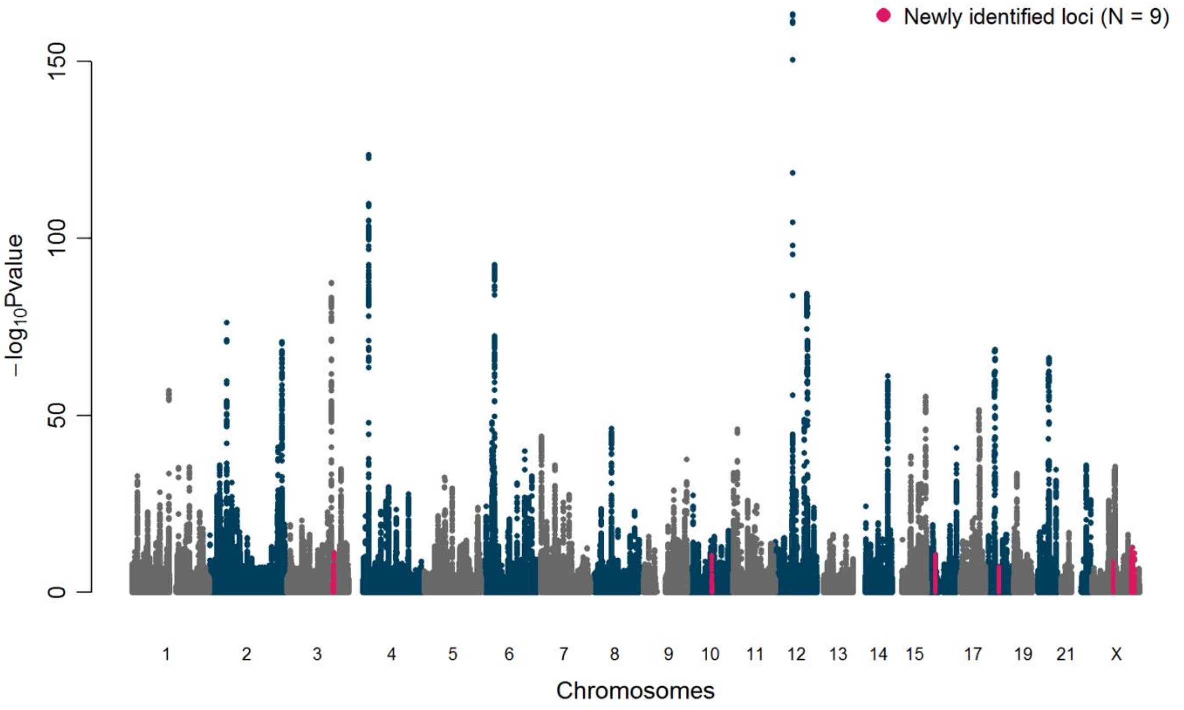

b

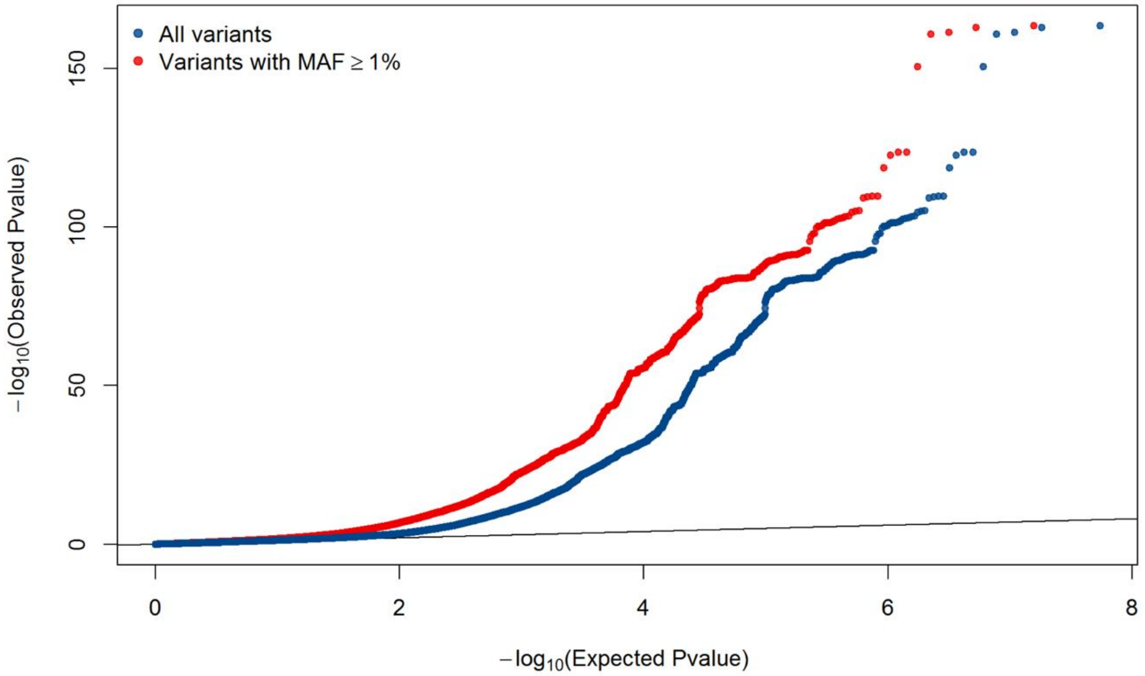

Supplementary Figure 3 | Manhattan plot and QQ plot of GWAS for human height

The Manhattan plot of GWAS (N = 159,195) is shown (a). QQ plot for all analyzed variant is colored blue, and for variants with minor allele frequency (MAF) ≥ 1% is colored red (b).

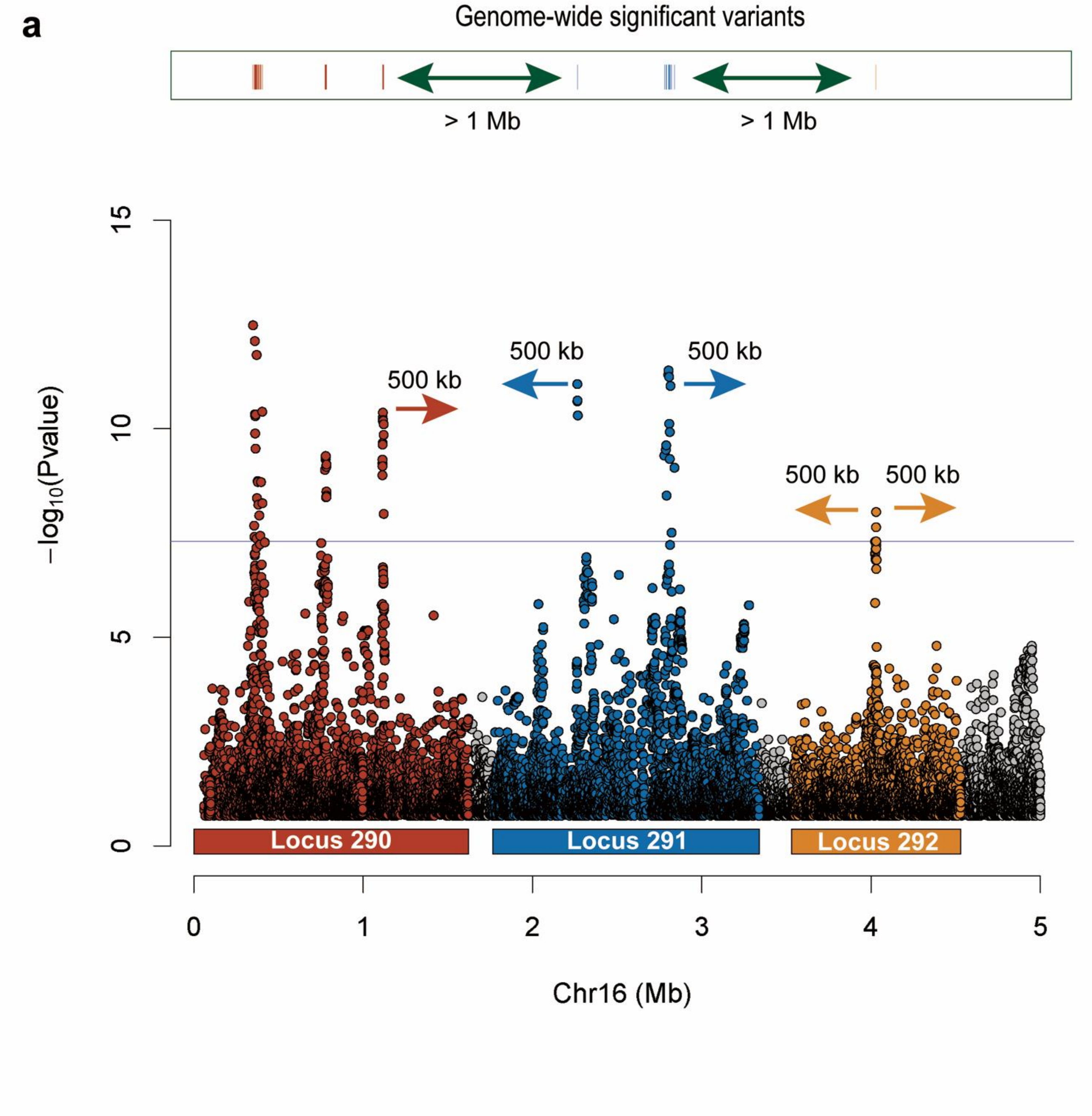
a
Genome-wide significant variants
> 1 Mb
> 1 Mb
500 kb
500 kb
500 kb
500 kb
500 kb
−log10(Pvalue)
15
10
5
0
Locus 290
Locus 291
Locus 292
0
1
2
3
4
5
Chr16 (Mb)

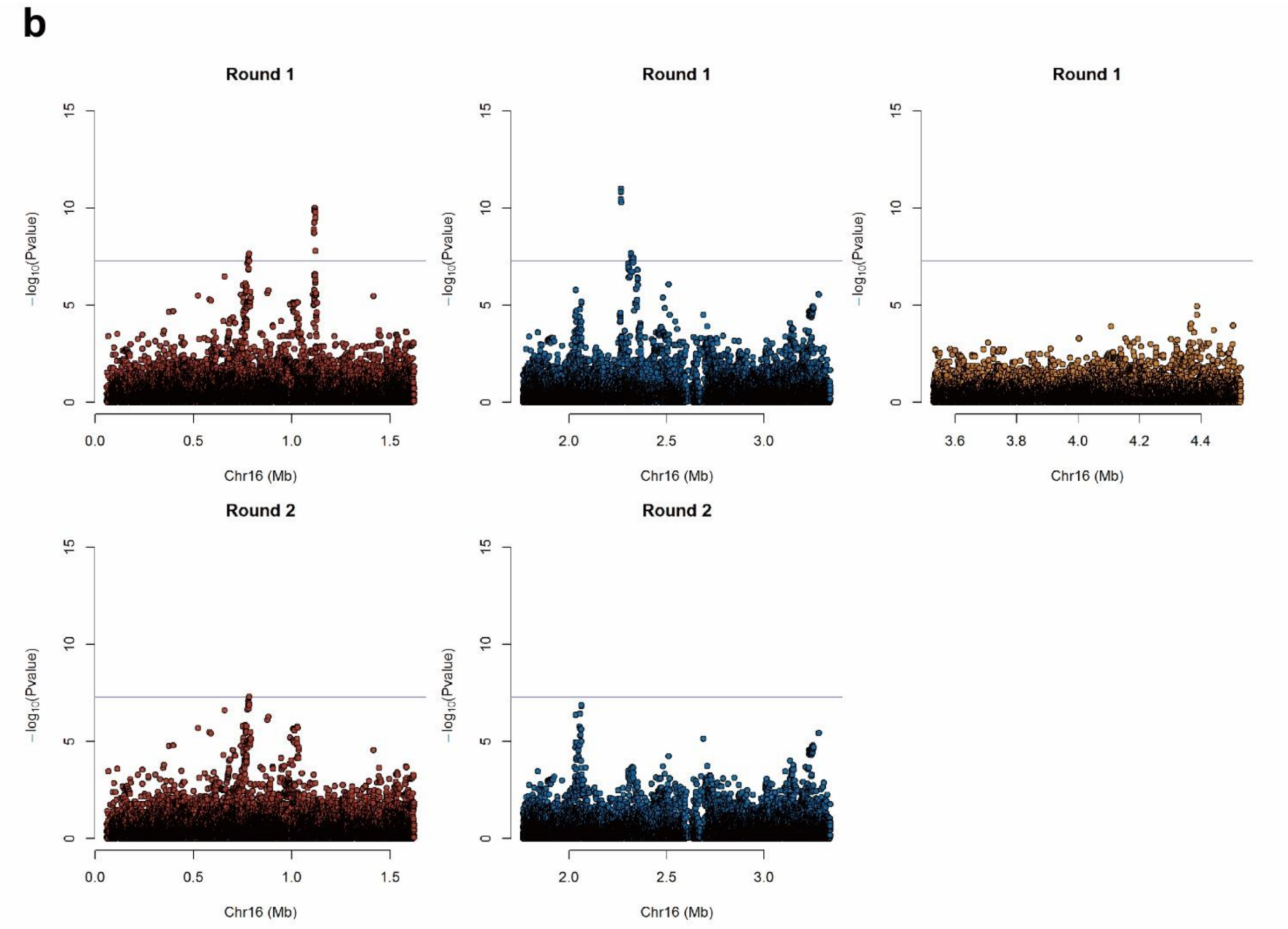

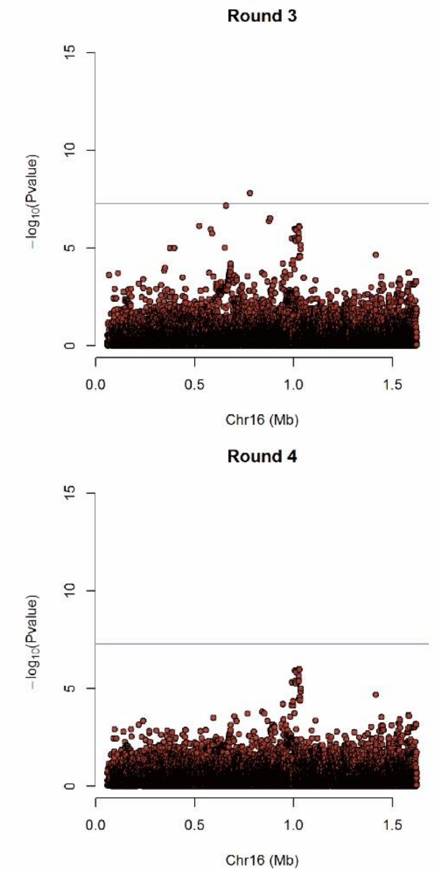

Supplementary Figure 4 | Definition of the locus and the strategy of conditional analysis.
To define the significantly associated loci, we first considered the positional relationship of the variants that reached genome-wide significant level of association (a: upper box). In case such variants were more than 1 Mb apart from each other, we regarded them as independent. After that, we extended the region by adding 500 kb on both sides (a: arrows in the regional plot), and carried out stepwise conditional analyses to determine independently associated variants (b).

In the examples depicted here (Locus 290-292), we determined three loci, and detected four, two, and one independently associated variants at Locus 290, 291 and 292, respectively.

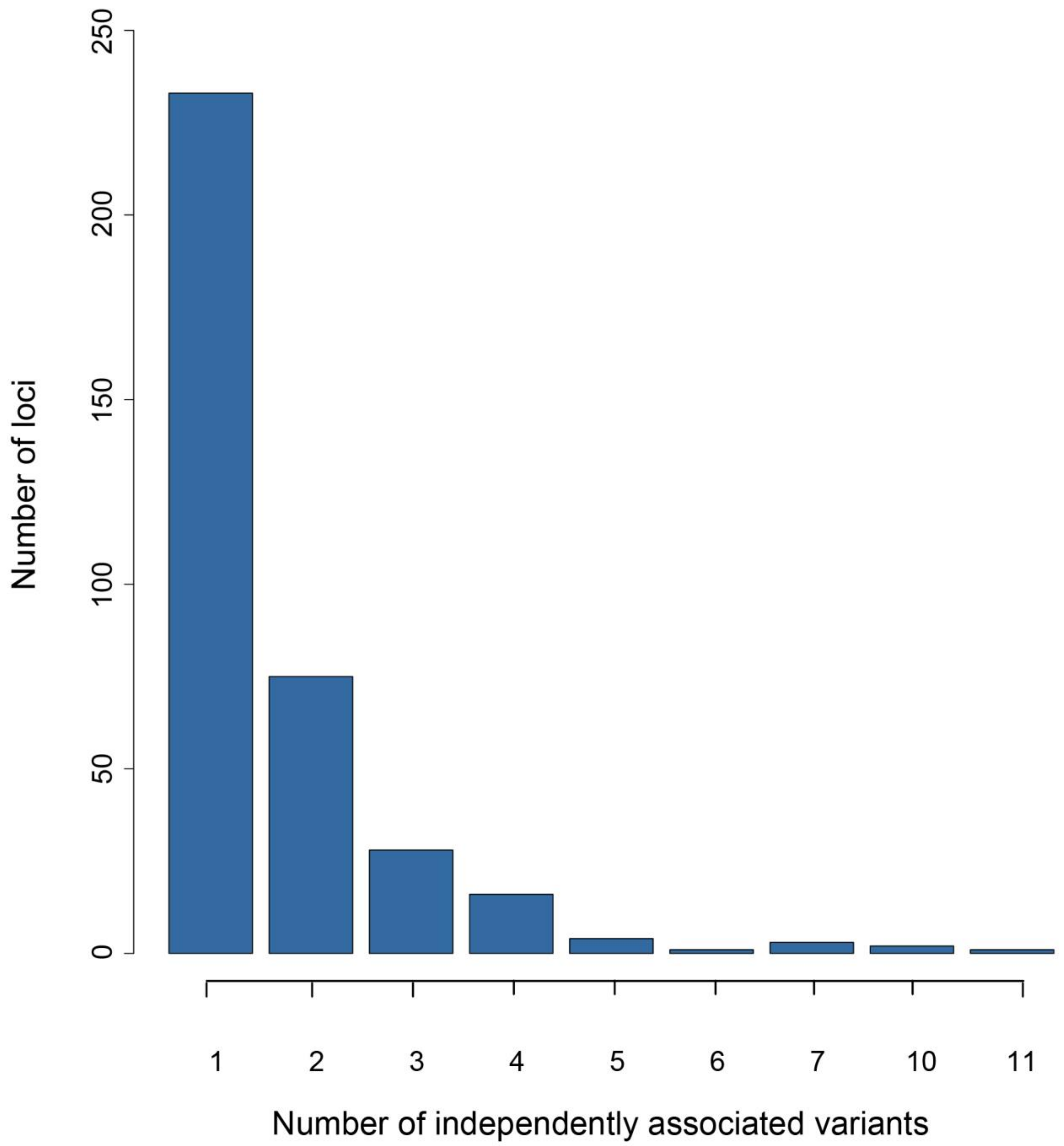

Supplementary Figure 5 | Number of the independently associated variants in height-associated loci

We performed conditional analysis and detected 246 independently associated variants within 130 identified loci in GWAS. The bar plot shows the number of associated variants identified in 363 identified loci.

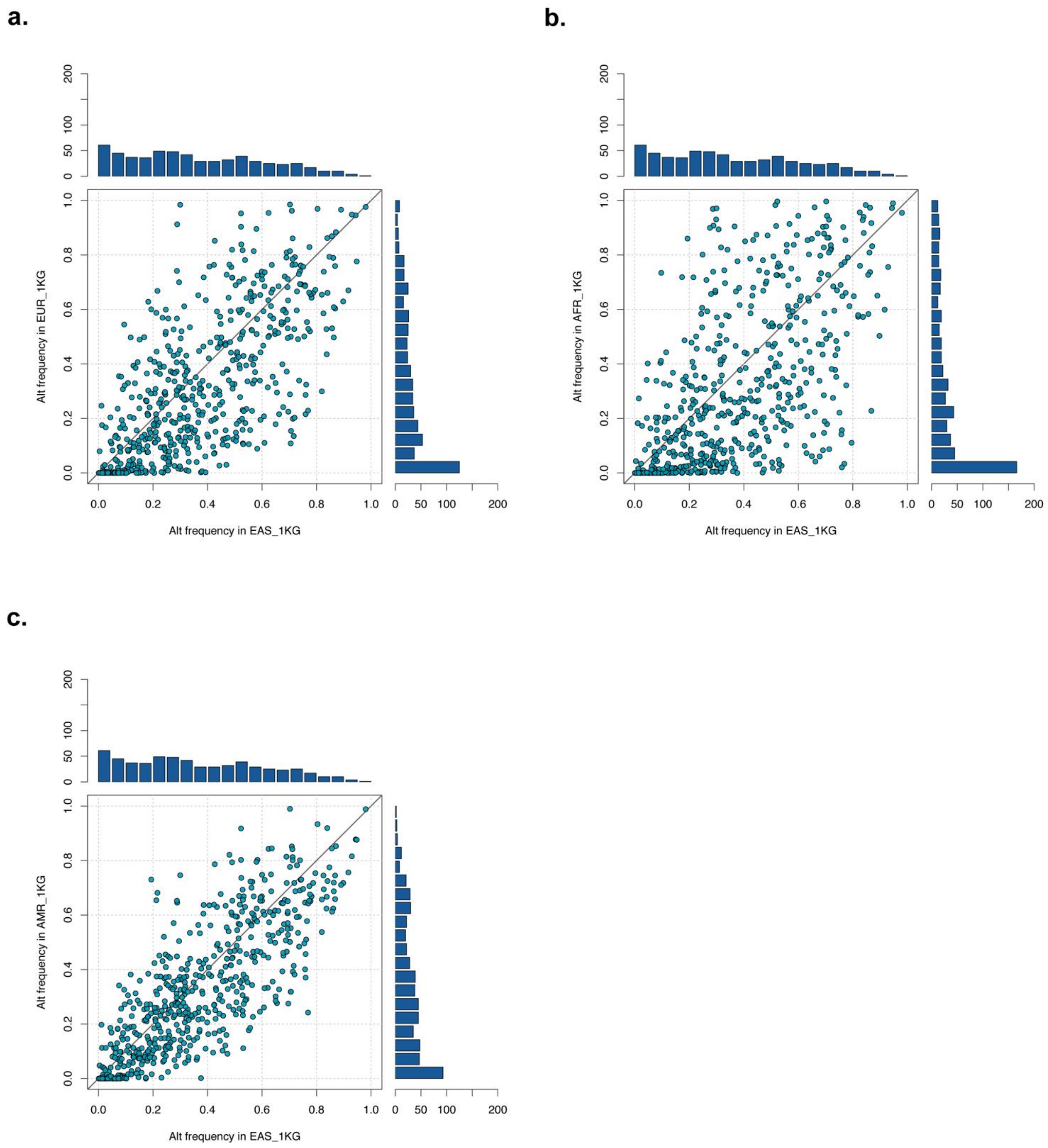

Supplementary Figure 6 | Trans-ethnic comparisons of the allele frequency of height-associated variants in the 1000 genomes project

We compared the allele frequency of identified variants between different ethnic groups. Among the 609 significant variants in GWAS, 591 were called in the 1000 genomes project. We plotted alternative allele frequency of these variants between East Asian (EAS) and European (EUR) (a), EAS and African (AFR) (b), and EAS and Ad Mixed American (AMR) (c), respectively. Histograms show counts of variants in each allele frequency strata.

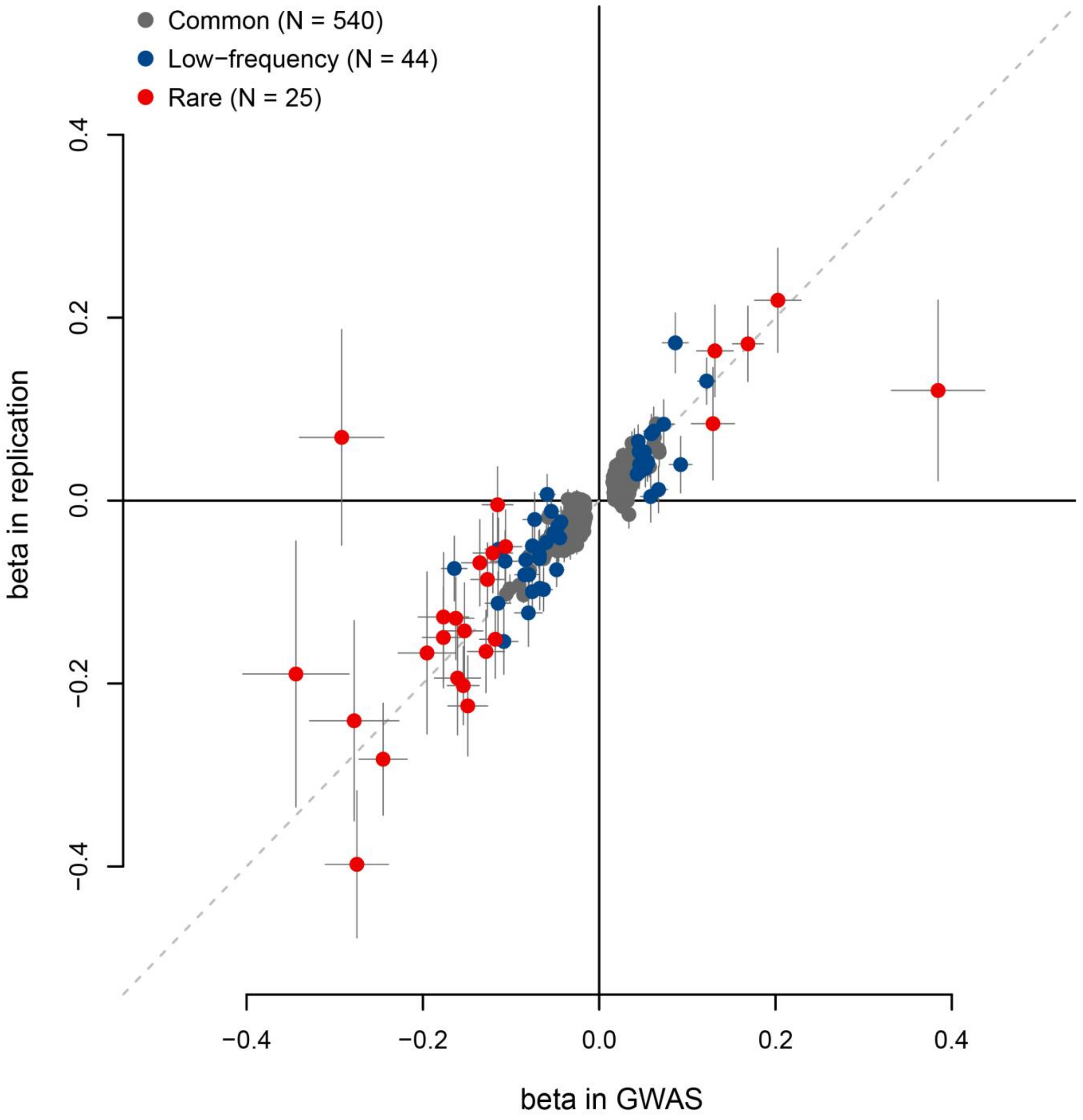

Supplementary Figure 7 | Comparison of effect sizes between GWAS and replication
Effect sizes of 609 identified variants in GWAS and replication are shown. Error bars denote standard error. Variants were colored according to their minor allele frequencies (red: MAF < 1%, blue: 1 ≤ MAF < 5%).

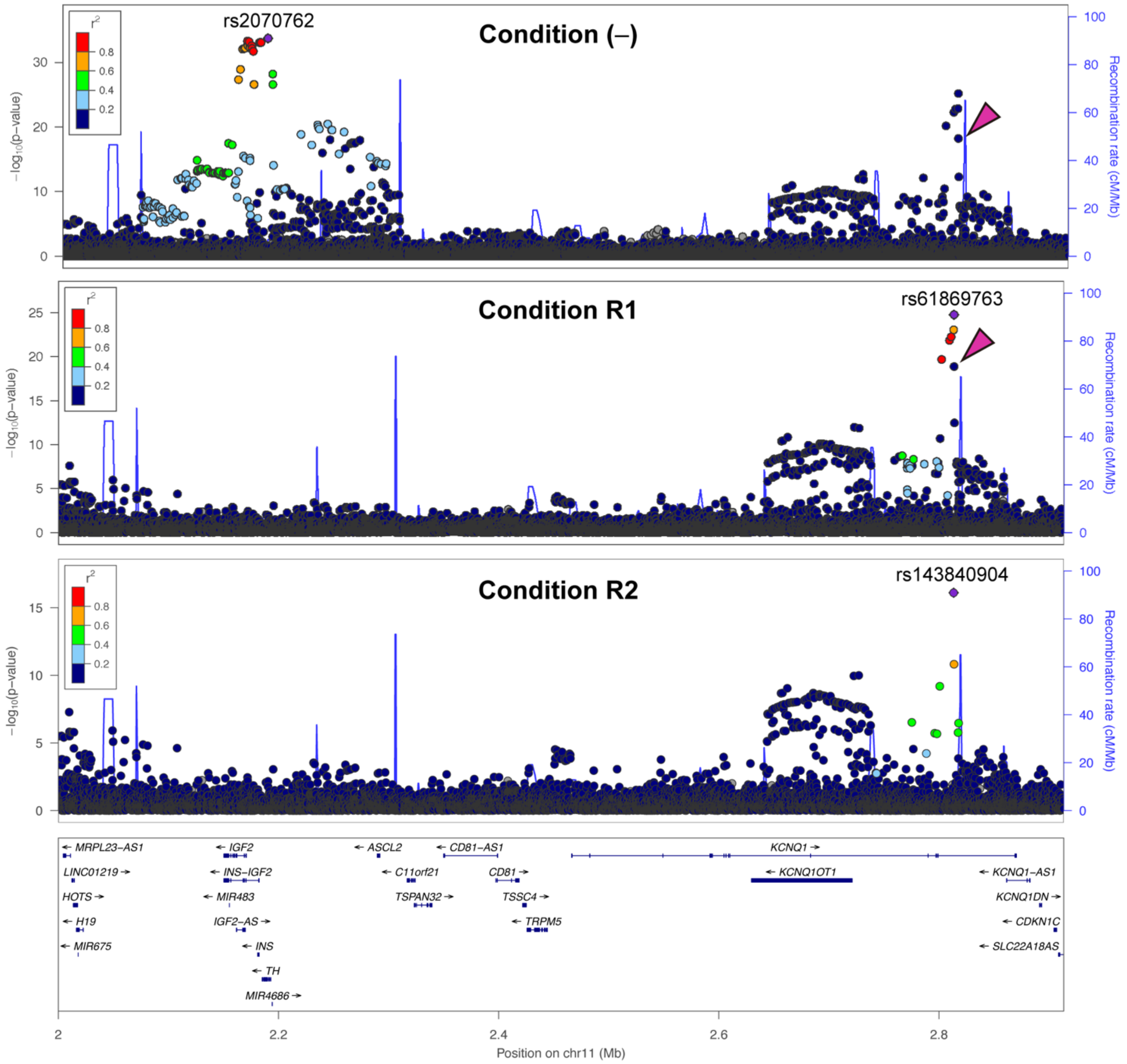

Supplementary Figure 8 | Regional association plot of *KCNQ1* locus

Regional association plot of *KCNQ1* locus (locus 215). The associations of GWAS (not conditioned) was plotted in the upper box. The plots of middle box indicate the associations of variants which were conditioned on rs2070762. The lower box illustrates the associations of variants conditioned by rs2070762 and rs61969763. The purple triangles denote rs143840904.

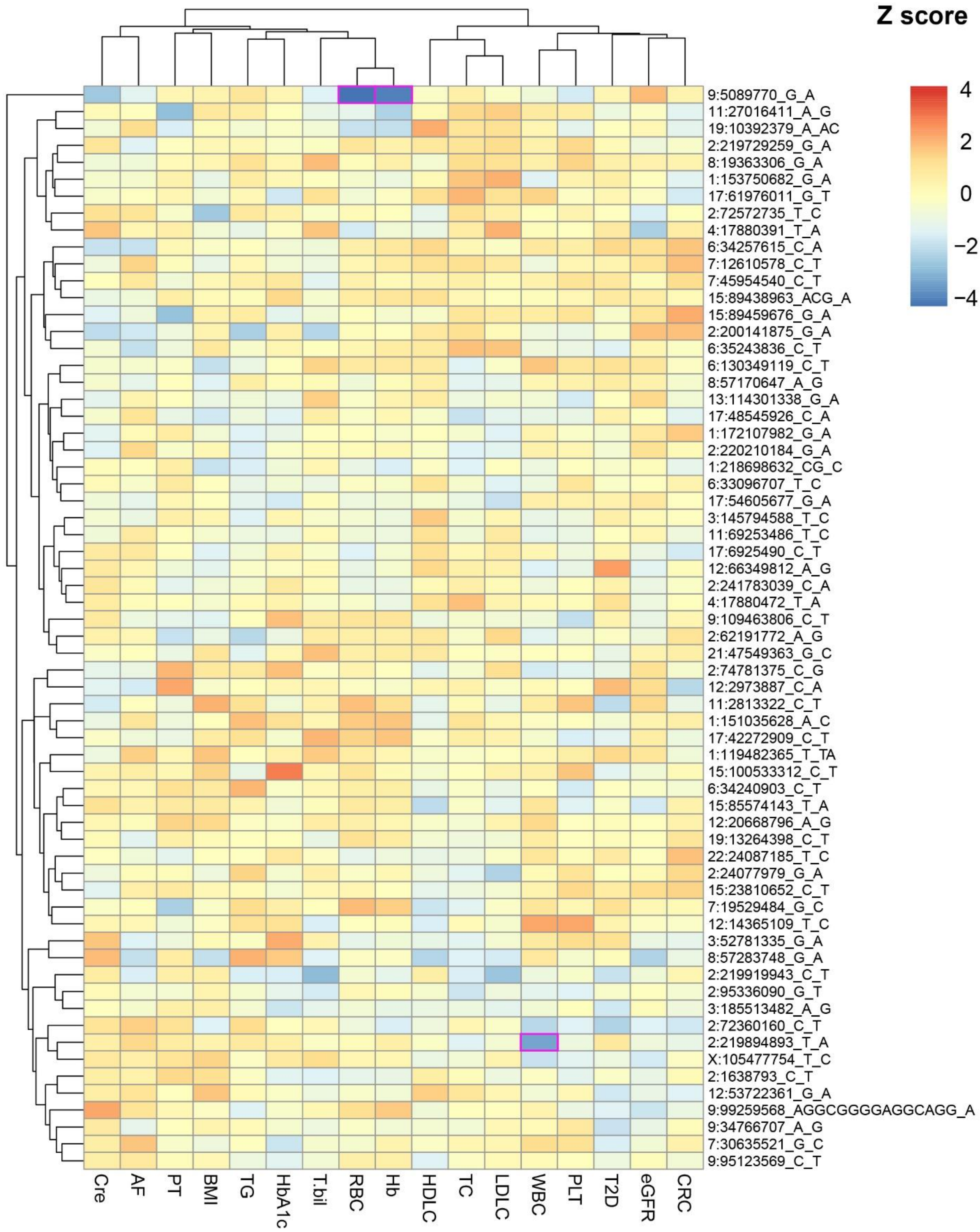

Supplementary Figure 9 | Pleiotropic associations

We evaluated the pleiotropic effects of 64 rare and low-frequency height-associated variants across 17 traits. The heatmap shows z score of each pleiotropic association. AF, atrial fibrillation; T2D, type 2 diabetes; CRC, colorectal cancer.

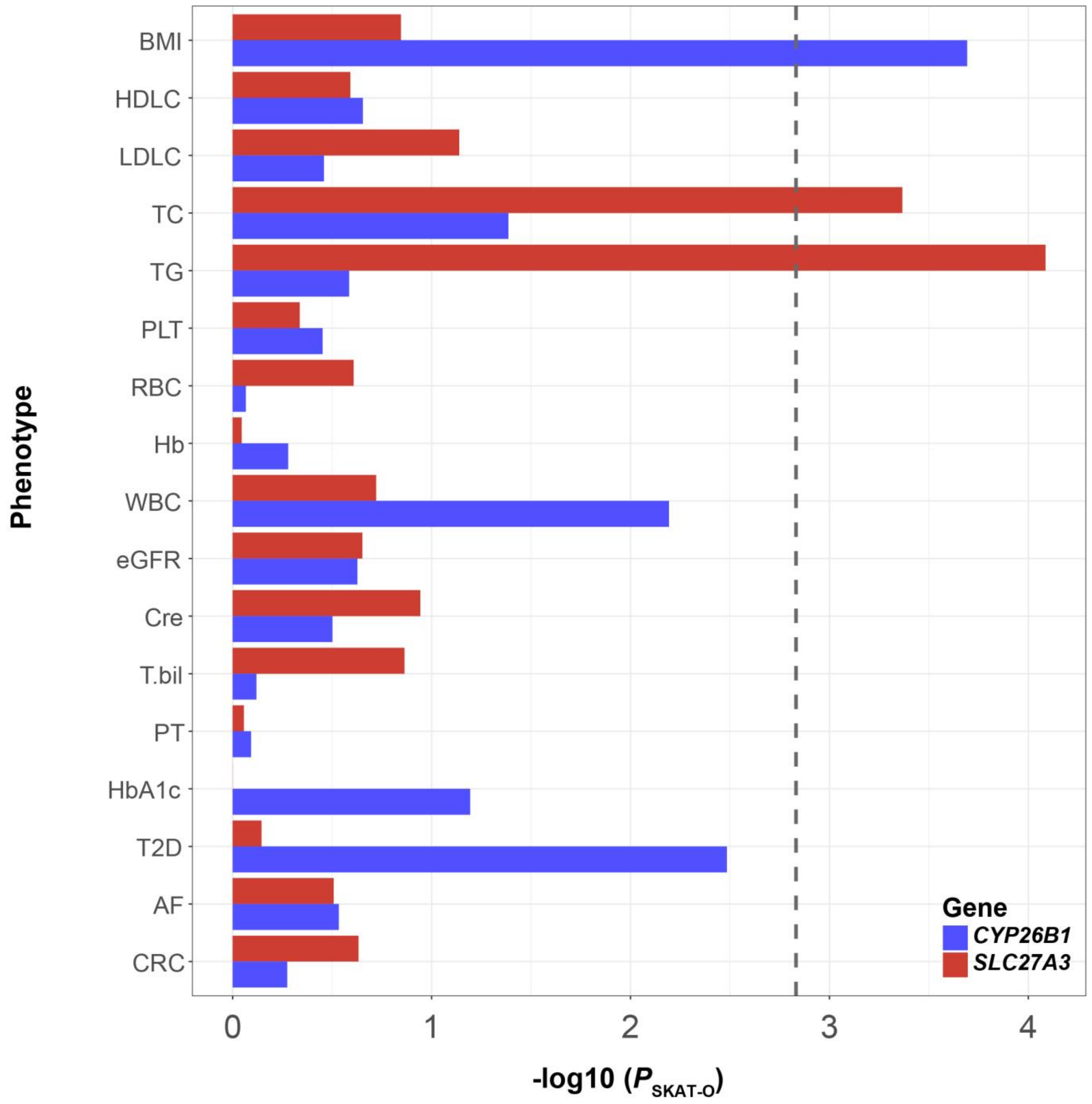

Supplementary Figure 10 | Pleiotropy of *SLC27A3* and *CYP26B1*

We evaluated pleiotropic associations of SLC27A3 (red) and CYP26B1 (blue) across 17 traits. Each bar indicates strength of association in -$\log_{10}$(P) scale. Dashed line denotes significant threshold after Bonferroni correction (α = 0.05/(17×2)).

T2D, type 2 diabetes; AF, atrial fibrillation; CRC, colorectal cancer.

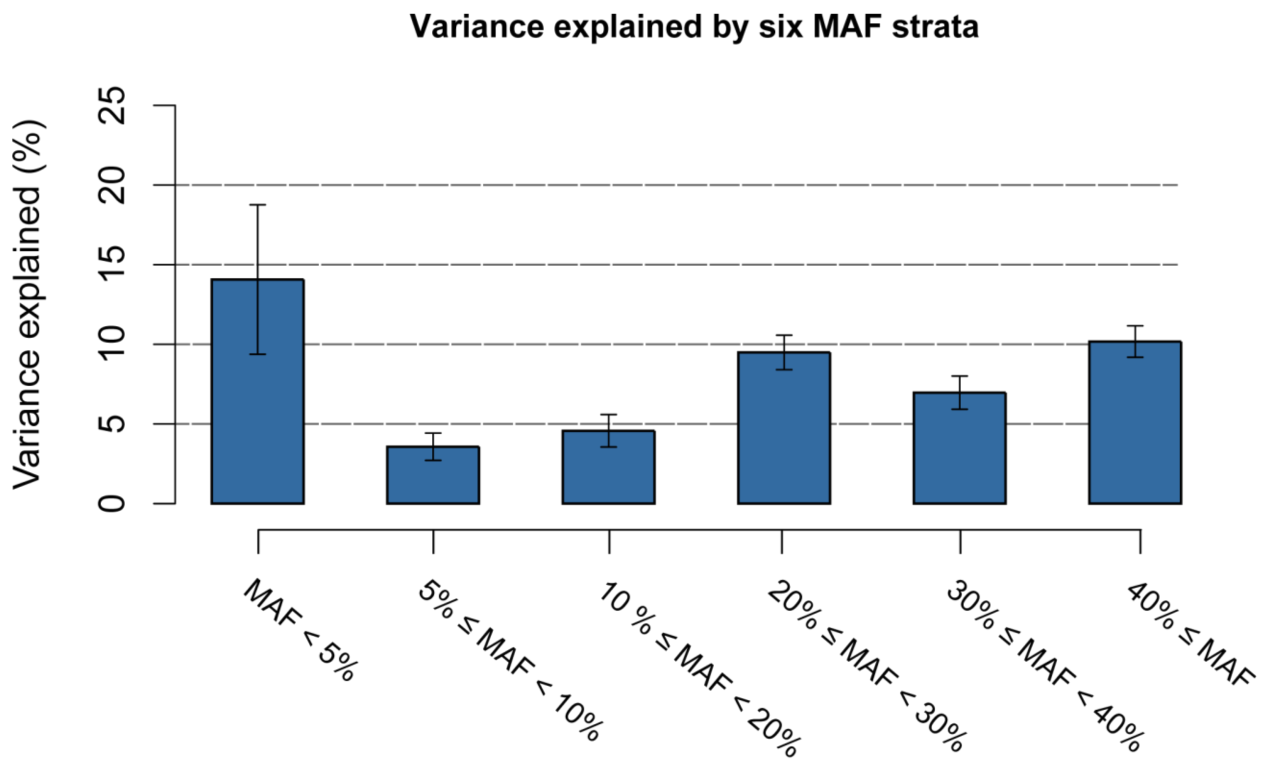

Supplementary Figure 11 | Variance explained by variants stratified by MAF
We estimated the variance explained by autosomal variants using GREML-MS using GCTA (ver 1.25). Explained variances of variants stratified according to minor allele frequency (MAF) into six strata are shown as bar plots. Error bars denote standard errors.

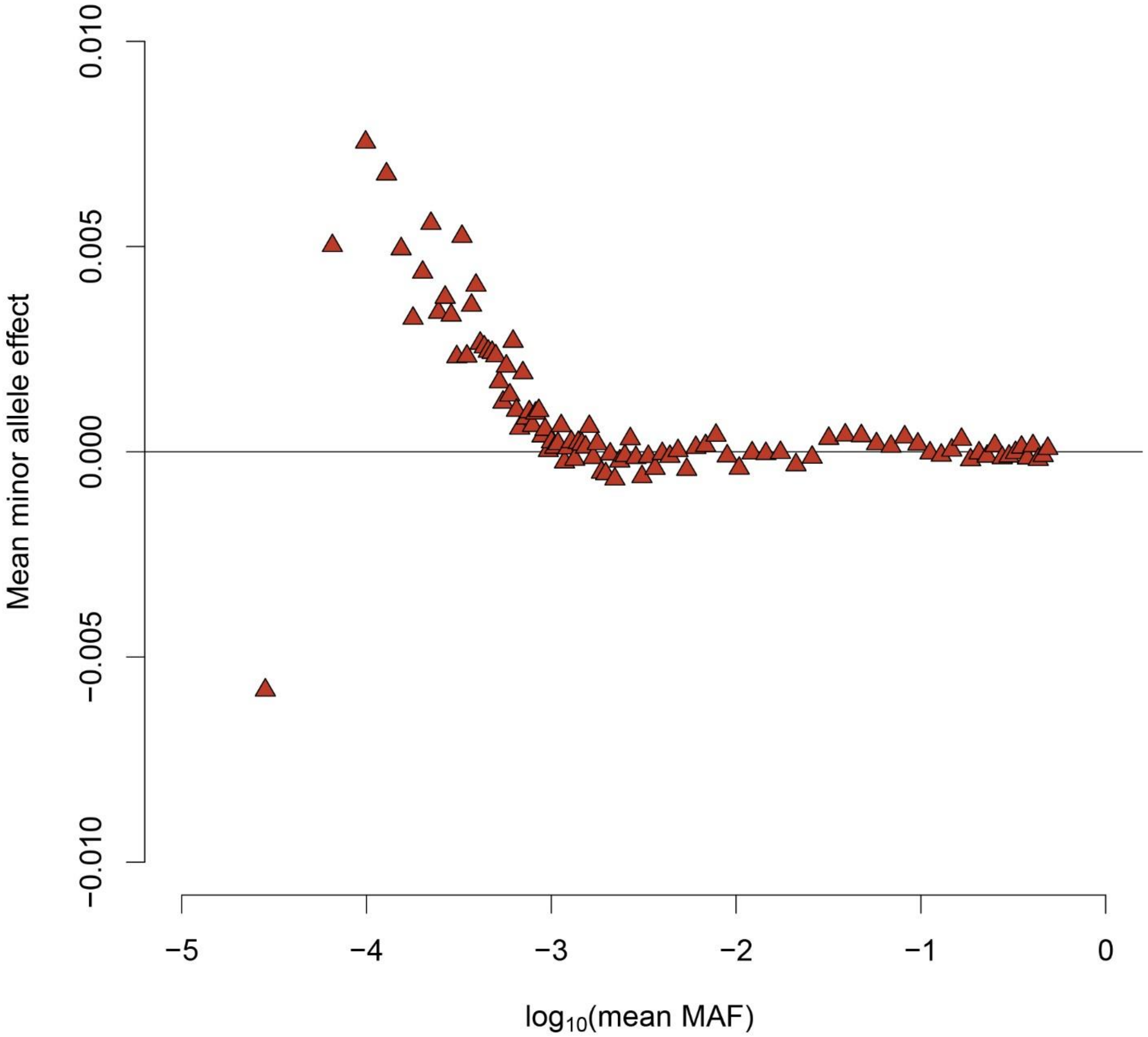

Supplementary Figure 12 | The relationship between minor allele effect and MAF in the replication set

Scatter plot of the minor allele effect against minor allele frequency in the replication set (Japanese population-based cohorts; *N* = 32,692).

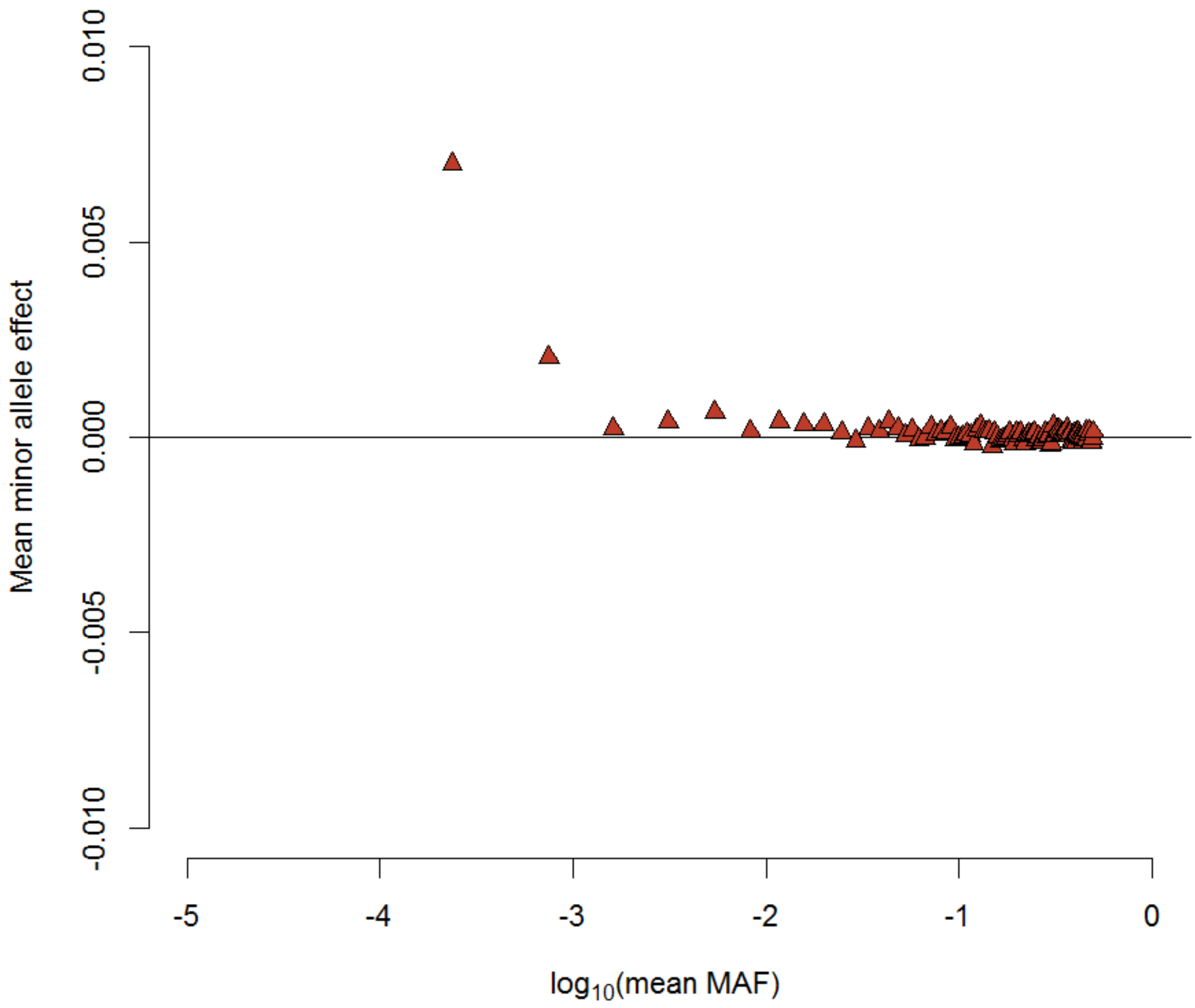

Supplementary Figure 13 | The relationship between minor allele effect and MAF of directly genotyped variants in the GWAS

Scatter plot of the minor allele effect against minor allele frequency of the directly genotyped variants in the GWAS.

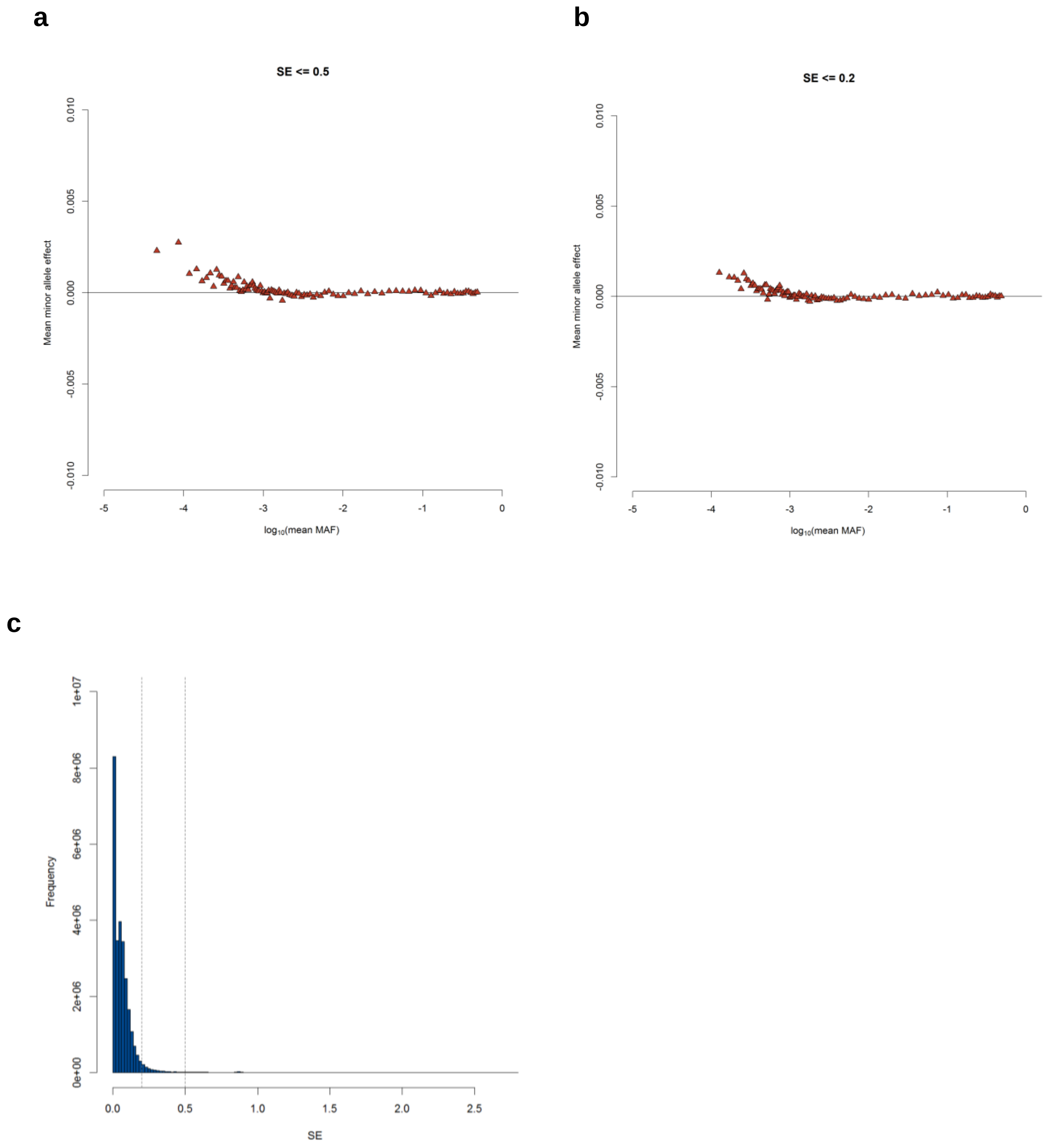

Supplementary Figure 14 | The relationship between minor allele effect and MAF of variants restricted by the same threshold of standard errors

Scatter plot of the minor allele effect against minor allele frequency of the variants with standard errors of effect sizes less than and equal to 0.5 (a), and less than and equal to 0.2 (b) in GWAS. These thresholds were determined by visual inspection of the histogram of standard errors of estimates (c).

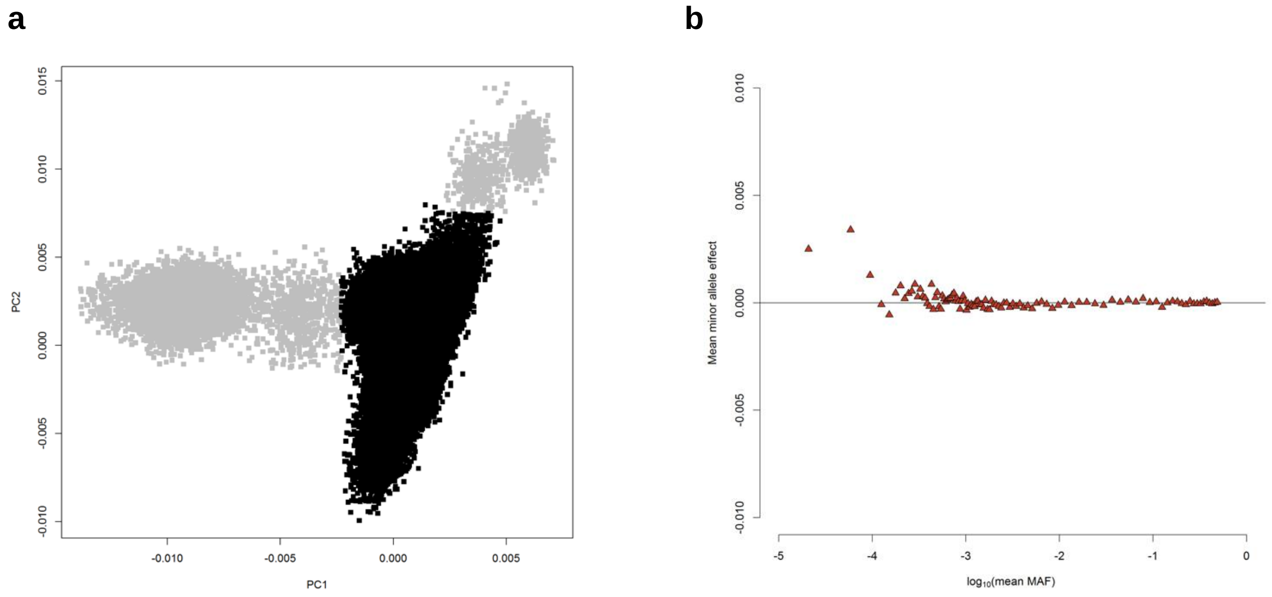

Supplementary Figure 15 | Stratified analysis using the samples of Japanese main cluster

To assess the impact of population stratification on the analysis on the relationship between minor allele frequency and minor allele effect, we selected the individuals who belonged to Japanese main cluster (a; *N* = 148,937, shown as black dots), and performed GWAS. Using the statistics of the GWAS, we evaluated the relationship between minor allele frequency and minor allele effect (b).

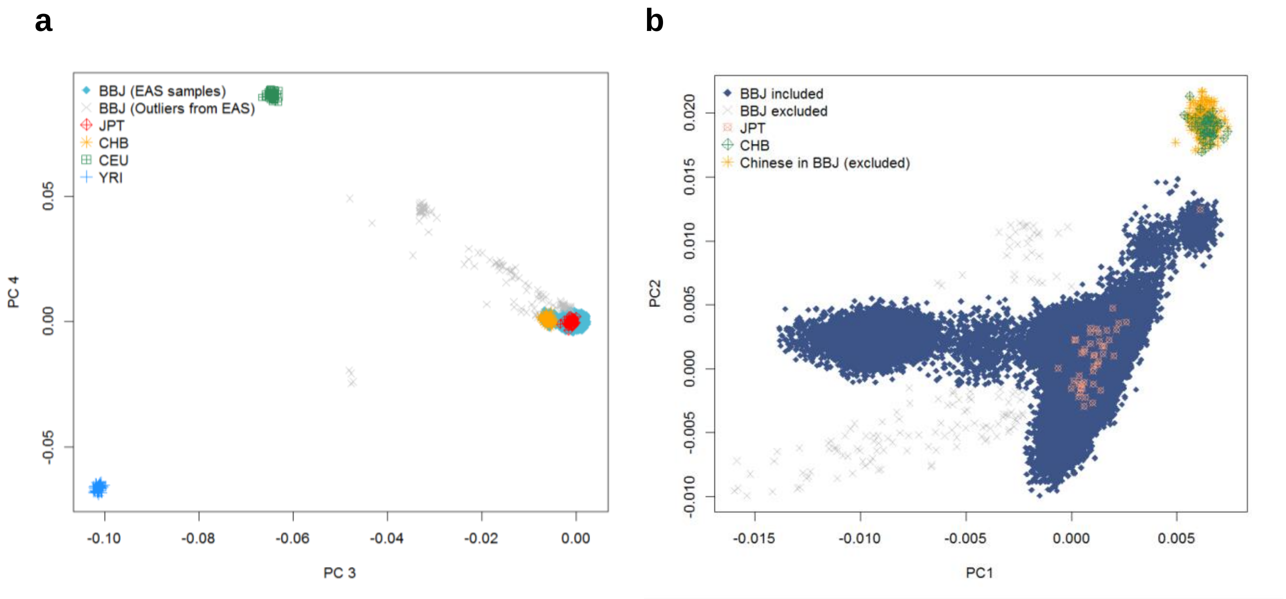

Supplementary Figure 16 | PCA plot

To exclude outliers of principle component analysis (PCA), we performed PCA of genotypes of the participants of BBJ with samples of HapMap project as references. We first performed PCA using HapMap four populations (CEU, YRI, JPT, and CHB) and determined outliers from East Asian population (a). After that, we re-performed PCA using East Asian samples (JPT, and CHB) and excluded samples which did not belong to the Japanese cluster.

Supplementary Tables:

Supplementary Table 1. Baseline characteristics of participants

Stage	Study	Male			Female		
		N	Age [year; mean±SD]	Height [cm; mean±SD]	N	Age [year; mean±SD]	Height [cm; mean±SD]
GWAS	BBJ	86,257	63.3 ± 13.2	165.4 ± 6.6	72,838	62.2 ± 14.8	152.9 ± 6.4
Replication	IMM	1,807	64.0 ± 9.7	164.5 ± 6.5	3,187	62.2 ± 10.2	152.1 ± 6.0
	JPHC	3,200	54.1 ± 8.2	162.6 ± 6.3	5,920	53.8 ± 7.8	151.1 ± 5.4
	JMICC	6,345	55.4 ± 9.3	167.3 ± 6.4	7,714	54.4 ± 9.4	154.7 ± 5.8
	TMM	1,438	62.3 ± 10.8	165.7 ± 6.3	3,081	58.2 ± 12.1	154.0 ± 5.8

Supplementary Table 2. Lead variants of the eight X chromosomal loci.

rsID	Candidate gene(s)	Locus	CHR:POS[a]	REF/ALT	Male (N = 86,257)		Female (N = 72,838)		Sex-combined analysis (N = 159,095)				
					BETA (SE)[b]	*P*	BETA (SE)[b]	*P*	Alt freq	MAF	BETA (SE)[b]	P_{meta}	P_{het}
rs7058676	*FAAH2*	Locus356	X:57348523	C/T	-0.036 (0.004)	3.09E-21	-0.029 (0.006)	4.11E-07	0.295	0.295	-0.034 (0.003)	1.16E-26	0.346
rs4844299	*BCYRN1*	Locus357	X:70854274	T/C	0.019 (0.004)	4.08E-08	0.013 (0.005)	1.32E-02	0.548	0.452	0.017 (0.003)	2.70E-09	0.352
rs5938929	*MIR325,FGF16*	Locus358	X:76436875	G/C	-0.040 (0.004)	4.59E-25	-0.043 (0.006)	6.44E-13	0.270	0.270	-0.041 (0.003)	2.44E-36	0.701
rs140498234	*MUM1L1,CXorf57*	Locus359	X:105477754	T/C	0.061 (0.012)	5.90E-07	0.083 (0.019)	9.44E-06	0.026	0.026	0.068 (0.010)	3.96E-11	0.333
rs3848873	*SLC25A43,SLC25A5-AS1*	Locus360	X:118594422	T/C	0.025 (0.003)	3.85E-13	0.022 (0.005)	2.23E-05	0.588	0.412	0.024 (0.003)	4.54E-17	0.666
rs148300465	*RAB33A,ZNF280C*	Locus361	X:129325425	T/C	0.043 (0.008)	7.28E-08	0.055 (0.012)	5.39E-06	0.071	0.071	0.047 (0.007)	2.60E-12	0.401
rs7065171	*LINC00629*	Locus362	X:133683590	C/G	0.024 (0.004)	3.91E-08	0.035 (0.007)	4.51E-07	0.788	0.212	0.027 (0.004)	1.88E-13	0.214
rs426349	*LOC389895,SOX3*	Locus363	X:139282091	C/T	0.025 (0.004)	2.49E-10	0.017 (0.006)	4.92E-03	0.502	0.502	0.023 (0.003)	8.12E-12	0.274

[a]Chromosomes and positions are based on Build37 (hg19).

[b]Alternative alleles were treated as effect alleles.

Linear regression analysis was performed using mach2qtl.

Supplementary Table 3. Associations at variants reported by GIANT consortium.

rsID	CHRa	POSa	Ref/Alt	Gene	BBJ GWAS (*N* = 159,095)			GIANT consortium (discovery+validation)			
					MAF	BETA (SE)	*P* value	*N*	MAF	BETA (SE)	*P* value
rs61730011	1	119,427,467	A/C	*TBX15*	0.001	-0.014 (0.063)	8.60E-01	621,267	0.044	-0.058 (0.005)	2.79E-36
rs145659444	1	149,902,342	C/T	*NSTNSR11*	0.001	-0.089 (0.114)	2.40E-01	602,837	0.007	0.073 (0.012)	3.03E-10
rs11580946	1	150,551,327	G/A	*NSCL1*	0.001	-0.068 (0.103)	7.70E-01	612,116	0.015	0.070 (0.008)	1.55E-19
rs141845046	1	154,987,704	C/T	*ZBTB7B*	0.0001	0.085 (0.245)	4.70E-01	634,019	0.027	0.059 (0.006)	3.46E-25
rs79485039	1	180,886,140	C/T	*KIAA1614*	0.003	-0.013 (0.040)	8.40E-01	607,431	0.028	0.033 (0.006)	2.63E-09
rs144712473	1	183,495,812	A/G	*SNSG7*	0.0003	0.111 (0.144)	6.20E-01	630,334	0.007	-0.083 (0.011)	1.61E-14
rs16866412	2	179,474,668	G/A	*TTN*	0.134	0.000 (0.005)	7.80E-01	634,022	0.012	-0.042 (0.008)	3.44E-07
rs147445258	2	220,078,652	C/T	*ABCB6*	0.001	0.107 (0.077)	2.10E-01	572,046	0.010	-0.079 (0.010)	2.47E-15
rs76208147	3	47,162,886	C/T	*SETD2*	0.147	0.035 (0.004)	3.50E-18	623,795	0.018	0.053 (0.007)	1.65E-13
rs35713889	3	49,162,583	C/T	*LANSB2*	0.0003	-0.101 (0.103)	4.40E-01	608,288	0.041	0.050 (0.005)	3.49E-27
rs9838238	3	98,600,385	T/C	*DCBLD2*	0.017	-0.015 (0.012)	1.50E-01	633,565	0.048	0.028 (0.004)	1.68E-12
rs146301345	5	32,784,907	G/A	*AC026703.1*	0.001	0.030 (0.074)	6.70E-01	554,983	0.003	0.139 (0.019)	7.91E-14
rs34821177	5	126,250,812	C/T	*NSARCH3*	0.005	-0.021 (0.035)	7.20E-01	622,159	0.034	0.032 (0.005)	1.67E-10
rs78247455	5	176,722,005	G/A	*NSD1*	0.014	-0.007 (0.013)	5.20E-01	625,766	0.024	-0.084 (0.006)	2.32E-41
rs33966734	6	41,903,798	C/A	*CCND3*	0.000	-0.141 (0.236)	7.20E-01	329,984	0.012	-0.122 (0.012)	1.28E-22
rs148543891	6	155,450,779	A/G	*TIANS2*	0.001	0.031 (0.074)	7.50E-01	527,454	0.003	-0.117 (0.021)	3.96E-08
rs11575580	9	34,660,864	C/T	*IL11RA*	0.002	0.066 (0.050)	2.20E-01	634,036	0.018	-0.050 (0.007)	4.01E-13
rs921122	9	95,063,947	C/T	*NOL8*	0.050	0.039 (0.007)	8.30E-08	406,442	0.040	0.029 (0.006)	3.33E-06
rs41274586	10	79,580,976	G/A	*DLG5*	0.002	0.052 (0.056)	2.90E-01	625,983	0.017	-0.065 (0.007)	7.66E-20
rs41291604	10	97,919,011	A/G	*ZNF518A*	0.043	0.007 (0.008)	5.20E-01	633,951	0.040	0.028 (0.005)	3.91E-09

rs71455793	11	65,715,204	G/A	*TSGA10IP*	0.000	-0.005 (0.084)	9.40E-01	634,057	0.042	-0.064 (0.005)	1.52E-43
rs4072796	12	7,548,996	C/G	*CD163L1*	0.156	0.023 (0.004)	6.40E-08	627,797	0.036	0.027 (0.005)	1.87E-08
rs117801489	12	104,408,832	T/C	*GLT8D2*	0.003	0.056 (0.027)	2.90E-02	633,998	0.022	0.057 (0.007)	1.60E-17
rs13141	12	121,756,084	G/A	*ANAPC5*	0.001	0.058 (0.080)	3.70E-01	630,220	0.010	-0.091 (0.010)	1.45E-21
rs117295933	14	45,403,699	C/A	*KLHL28*	0.003	-0.085 (0.031)	2.50E-03	582,555	0.020	-0.041 (0.007)	3.05E-09
rs34815962	15	72,462,255	C/T	*GRANSD2*	0.024	0.002 (0.010)	7.10E-01	612,199	0.021	0.073 (0.007)	1.28E-27
rs113388806	16	24,804,954	A/T	*TNRC6A*	0.001	0.174 (0.064)	1.30E-02	589,097	0.043	0.038 (0.005)	1.90E-15
rs8052655	16	67,409,180	G/A	*LRRC36*	0.002	0.126 (0.044)	4.60E-03	618,357	0.043	-0.054 (0.005)	6.40E-31
rs149615348	16	84,900,645	G/A	*CRISPLD2*	0.002	0.022 (0.036)	8.80E-01	630,065	0.008	-0.096 (0.011)	2.92E-19
rs77169818	18	74,980,601	A/T	*GALR1*	0.075	-0.031 (0.006)	1.10E-06	595,884	0.044	-0.044 (0.005)	5.11E-19

We looked up the variants identified by the recent exome array analysis conducted by GIANT consortium (Eirini et al. Nature 2017).

[a]Chromosomes and positions are based on Build37 (hg19).

Supplementary Table 4. Associations at reported rare and low-frequency variants.

rsID	CHR[a]	POS[a]	REF/ALT	ALT freq	BETA (SE)	P (BOLT-LMM)	Report	PMID
rs183677281	1	218,537,632	T/C	0.00082	-0.016 (0.086)	9.60E-01	Tachmazidou et al. Am J Hum Genet. 2017	28552196
rs202238847	3	49,263,637	CT/C	2.48E-06	-0.708 (1.840)	8.00E-01	Tachmazidou et al. Am J Hum Genet. 2017	28552196
rs142854193	7	33,045,510	C/T	0.02259	-0.008 (0.011)	5.50E-01	Tachmazidou et al. Am J Hum Genet. 2017	28552196
rs149658560	11	2,767,262	G/A	0.26090	-0.005 (0.004)	1.40E-01	Zoledziewska et al. Nat Genet 2015	26366551
rs67004488	11	2,787,804	A/G	0.20966	-0.011 (0.004)	3.80E-03	Zoledziewska et al. Nat Genet 2015	26366551
rs2075870	11	2,790,019	G/A	0.20517	-0.011 (0.004)	6.70E-03	Zoledziewska et al. Nat Genet 2015	26366551
rs143840904	11	2,813,322	C/T	0.01564	-0.122 (0.014)	6.40E-19	Zoledziewska et al. Nat Genet 2015	26366551

[a] Chromosomes and positions are based on Build37 (hg19).

Supplementary Table 5. Human phenotype information on 13 genes containing height associated nonsynonymous variants.

Gene	Extremely phenotype related to height in human
ACAN	Spondyloepimetaphyseal dysplasia, aggrecan type (**MIM**:612813; short stature by bi-allelic loss of function mutations), Spondyloepiphyseal dysplasia, Kimberle (**MIM**: 608361; short stature by bi-allelic loss of function mutations), Short stature and advanced bone age, with or without early-onset osteoarthritis and/or osteochondritis dissecans (**MIM**: 165800; short stature byheterozygousc loss of function mutations)
ADAMTS17	Weill-Marchesani syndrome (**MIM**: 613195 , short stature)
CSGALNACT1	Mild skeletal dysplasia and joint laxity (short stature by bi-allelic loss of function mutations. Vodopiutz et al. *Hum Mut* 2017)
FBN2	Congenital contractural arachnodactyly (**MIM:** 121050; contractures, arachnodactyly, scoliosis, tall stature)
GDF5	Acromesomelic dysplasia, Hunter-Thompson type (**MIM**: 201250; short stature by bi-allelic loss of function mutations), Chondrodysplasia, Grebe type (**MIM**: 200700; short stature by bi-allelic loss of function mutations)
GH1	Growth hormon defeciency (**MIM**:262400, 612781, 173100; short stature), Kowarski syndrome (**MIM**: 262650; short stature)
GHR	Laron dwarfism (**MIM:**262500; short stature)
IHH	Acrocapitofemoral dysplasia (**MIM**: 607778; short stature), Brachydactyly, type A1 (**MIM**: 112500; short stature)
NPR2	Acromesomelic dysplasia, type Maroteaux (**MIM**: 602875; short stature by bi-allelic loss of function), Short stature with nonspecific skeletal abnormalities (heterozygous, **MIM** 616255), overgrowth syndrome (heterozygous gain of function. See Miura et al. *PLoS One* 2012)
NSD1	Sotos syndrome 1 (**MIM:** 117550; excessively rapid growth and acromegalic features by heterozygous mutation)
PLOD2	Bruck syndrome 2 (**MIM**: 609220; short stature)
SP7 (Osterix)	Osteogenesis imperfecta, type 12 (**MIM**: 613849; short stature by bi-allelic loss of function mutation)
TRIP11	Achondrogenesis, type IA (**MIM:** 200600; short stature by bi-allelic loss of function mutations)

Supplementary Table 6. Traits analyzed in cross-phenotype analysis

a. Quantitative traits

phenotype	*N*	mean (SD)	Range		Unit	Inclusion criteria	Exclusion criteria	Statistical model to obtain residuals of linear regression
			Min	Max				
RBC	139,611	433.87 (56.36)	192.00	680.00	/µl	raw: +-3IQR	-	RAW ~ sex + age + age^2 + PC1-10
Hb	138,843	13.40 (1.79)	5.90	20.00	/µl	raw: +-3IQR	-	RAW ~ sex + age + age^2 + PC1-10
WBC	140,812	6216.64 (2080.63)	1480.00	23110.00	/µl	log transformation*: +-3IQR	-	log(RAW) ~ sex + age + age^2 + PC1-10
PLT	134,672	22.76 (7.24)	5.80	83.00	/µl	log transformation*: +-3IQR	-	RAW ~ sex + age + age^2 + PC1-10
TG	103,142	140.73 (97.90)	9.00	1514.00	mg/dl	log transformation*: +-3IQR	-	RAW ~ sex + age + age^2 + chol_drug + PC1-10
TC	125,223	199.16 (38.98)	21.00	376.00	mg/dl	raw: +-3IQR	-	RAW ~ sex + age + age^2 + chol_drug + PC1-10
HDLC	70,689	53.90 (14.28)	14.20	93.80	mg/dl	raw: +-1.5IQR**	-	RAW ~ sex + age + age^2 + chol_drug + PC1-10
LDLC	69,129	117.09 (31.02)	30.30	203.60	mg/dl	raw: +-1.5IQR**	-	RAW ~ sex + age + age^2 + chol_drug + PC1-10
BMI	158,025	23.27 (3.65)	10.50	61.30	kg/m^2	-	-	log(RAW)~ sex + age + age^2 + PC1-10 + 47 disease status
Cre	138,202	0.79 (0.27)	0.18	3.03	mg/dl	log transformation*: +-3IQR	Hemodialysis	RAW ~ sex + age + age^2 + PC1-10
eGFR	137,635	73.77 (20.81)	20.73	245.36	ml/min/1.73^2	log transformation*: +-3IQR	-	RAW ~ sex + age + age^2 + PC1-10
HbA1c	42,267	5.50 (0.61)	3.00	7.80	%	raw: +-3IQR	Diabetes	RAW ~ sex + age + age^2 + PC1-10
TBil	108,018	0.61 (0.29)	0.01	1.96	mg/dl	raw: +-3IQR	Hepatic diseases	RAW ~ sex + age + age^2 + PC1-10
PT	42,938	11.79 (1.22)	8.20	17.60	sec	raw: +-3IQR	Hepatic diseases, oral anticoagulant comsumption	RAW ~ sex + age + age^2 + PC1-10

* A reason for log transformation before deciding outlier threshold was skewed distribution of raw values.

**A reason for adopting 1.5 IQR is because lower limits fell in negative value when adopting 3.0 IQR.

b. Diseases

Phenotype	*N*		Exclusion criteria	Covariates used for association analysis
	Case	Control		
Atrial fibrillation (AF)	5,641	153,256	-	sex, age, PC1-10
Colorectal cancer (CRC)	5,480	153,417	-	sex, age, PC1-10
Type 2 diabetes (T2D)	29,404	126,306	Type 1 diabetes, maturity-onset diabetes of the young, mitochondrial diabetes	sex, age, PC1-10

Supplementary Table 7. Conditional analysis for *CYP26B1* and *SLC27A3*.

Gene	CHR	Best-associated nonsynonymous variant (amino acid change)	Crude		Conditioned on best-associated variant	
			N variants[a]	P_{SKAT-O}	N variants[a]	P_{SKAT-O}
SLC27A3	1	rs146128753 (p.G450R)	27	6.94×10^{-38}	26	3.38×10^{-25}
CYP26B1	2	rs138478634 (p.R323W)	17	1.33×10^{-45}	16	2.41×10^{-21}

Supplementary Notes:

Supplementary Note 1 | Reference panel construction and selection

a. Autosomes

We described the method to combine the whole-genome sequence data obtained from 1,037 Japanese individuals (BBJ1K) and that of 1000 genome project (phase3v5; N =2,504) in the online method. Finally, this reference panel contained 59,387,070 variants including 3,051,393 insertion and deletions. The accuracy of newly constructed reference panel for genotype imputation is shown in **Fig.1**.

b. X-chromosome

Since the current version of IMPUTE2 is not applicable to haploid chromosome, we evaluated six independent set of reference panel using the sample analyzed in GWAS as well as autosome. The evaluated reference panels were as follows: (1) EAS samples of 1000 genome project (phase1v3; N = 286). (2) ALL samples of 1000 genome project (phase3v5; N =2,504), (3) BBJ1K (N = 1,037), (4) BBJ1K + ALL samples of 1000 genome project (N = 3,541): we imputed missing genotypes in each data set using Beagle, and combined them. (5) BBJ1K + ALL samples of 1000 genome project (N = 3,541): after the process of (4), we phased haplotypes using SHAPEITv2, (6) BBJ1K + ALL samples of 1000 genome project (N = 3,541): we used IMPUTE2 to impute the missing value for female, and used Beagle for male, and merged. The result of imputation accuracies evaluated by mean true r^2 and number of variants satisfying certain imputation quality score (Rsq value) were shown in **Supplementary Fig.2a**.

We interpreted that using BBJ1K panel could improve imputation quality of X chromosomal variants. Although imputation accuracies of BBJ1K panel and BBJ1K + 1KG ALL panel were comparable, we observed that merged panel contains greater number of variants than BBJ1K (**Supplementary Fig.2b**). According to these results, we determined to use merged reference panel (6) for genotype imputation of X-chromosome.

Supplementary Note 2 | Descriptions of the participating cohorts

The Biobank Japan (BBJ)

The BBJ Project (http://biobankjp.org) started at the Institute of Medical Science, the University of Tokyo in 2003. To date, the BBJ Project has collected around 200,000 individuals with disease cases consisting of 47 various diseases. These subjects were recruited from 12 medical institutes in Japan including, Osaka Medical Center for Cancer and Cardiovascular Diseases, the Cancer Institute Hospital of Japanese Foundation for Cancer Research, Juntendo University, Tokyo Metropolitan Geriatric Hospital, Nippon Medical School, Nihon University School of Medicine, Iwate Medical University, Tokushukai Hospitals, Shiga University of Medical Science, Fukujuji Hospital, National Hospital Organization Osaka National Hospital, and Iizuka Hospital.

Japan Multi-Institutional Collaborative Cohort Study (J-MICC study)

In the Japan Multi-Institutional Collaborative Cohort (J-MICC) Study, 40,892 men and 51,750 women aged 35 to 69 years completed questionnaires on medical histories and donated blood samples at the baseline survey between 2004 and 2014. They were recruited in 14 study areas throughout Japan from community dwellers, first-visit patients in a cancer hospital, or health checkup examinees. For the present analyses, about 500 to 2,000 participants were selected from each study area, considering the number of respondents from each field and the geographical distribution of subjects. All the participants provided written informed consent. The ethics committees of Nagoya University (the affiliation of the principal investigator) and the other participating institutions approved the protocol for the J-MICC Study. The J-MICC Study was supported by Grants-in-Aid for Scientific Research for Priority Areas of Cancer (No. 17015018) and Innovative Areas (No. 221S0001) and JSPS KAKENHI Grant (No. 16H06277) from the Japanese Ministry of Education, Science, Sports, Culture and Technology. The following research institutions participated in the study; Chiba Cancer Center, University of Shizuoka, Nagoya City University, Aichi Cancer Center, Nagoya University, Shiga University of Medical Science, Tsuruga Nursing University, Kyoto Prefectural University of Medicine, University of Tokushima, Kyushu University, Saga University, and Kagoshima University.

Japan Public Health Center-based Prospective Study (JPHC)

The JPHC samples were derived from a cohort of 33,736 residents in 9 public health center (PHC) areas who not only returned a self-administered questionnaire but also donated 10 mL of venous blood at the baseline survey. For the first step of sample selection, we stratified the cohort by sex, 5-year age categories, and 9 PHC areas, and then conducted a random sampling, in which a similar proportion of subjects was selected from each stratum.

Consequently, we determined 9,296 subjects for the present GWAS. Before using the JPHC samples for genetic research, we obtained an approval from the institutional review board of the National Cancer Center (approval number: 2011-044), Tokyo, Japan, and provided all eligible subjects with the opportunity to refuse participation in the research.

The Tohoku Medical Megabank (TMM) Project

- **Iwate Tohoku Medical Megabank Organization (IMM)**
- **Tohoku Medical Megabank Organization (ToMMo)**

The TMM Project is a reconstruction project from the Great East Japan Earthquake, 2011, conducted by Tohoku University (http://www.megabank.tohoku.ac.jp/english/) and Iwate Medical University (http://iwate-megabank.org/en/)1. The TMM Project conducts two prospective cohort studies in Miyagi and Iwate Prefectures, Japan; the TMM Community-Based Cohort Study (TMM CommCohort Study) and the TMM Birth and Three-Generation Cohort Study (TMM BirThree Cohort Study). The TMM CommCohort Study is a population-based adult cohort study and has recruited approximately 84,000 participants aged 20 years or over during 2013–2016. The TMM BirThree Cohort Study has recruited around 74,000 participants including fetuses and their parents, siblings, grandparents, and extended family members as of July 2017. All participants in the TMM Project consent to genetic studies. biospecimens (blood and urine) and medical data (questionnaires, blood and urine tests, and physiological measurements) have been collected at baseline examination. These samples and information are stored in the integrated biobank of the TMM Project. DNA samples of the participants of the TMM CommCohort Study recruited in 2013 have been analyzed by using the Illumina OmniExpressExome array (N=10,000). Information about age and sex has been collected by using self-administered questionnaires and by reviewing municipal basic resident register. Of the 10,000 persons with genotype data, height and weight were measured for 9,202 persons in a standard manner. For persons without the measurement of body height and weight (N=798), these variables were obtained from self-reported questionnaires when available (N=703). Remaining 95 persons who had neither measured nor self-reported values are excluded from the analyses of the present study.
