# Supplementary material for: Characterizing rare and low-frequency height-associated variants in the Japanese population
Source: Nat Commun. 2019 Sep 27;10:4393. doi: 10.1038/s41467-019-12276-5 (PMC6764965; doi:10.1038/s41467-019-12276-5)
Supplement: Supplementary file 2 — Reporting Summary [file 41467_2019_12276_MOESM2_ESM.pdf]

nature research

Corresponding author(s): Yoichiro Kamatani

Reporting Summary

Nature Research wishes to improve the reproducibility of the work that we publish. This form provides structure for consistency and transparency in reporting. For further information on Nature Research policies, see Authors & Referees and the Editorial Policy Checklist.

Statistical parameters

When statistical analyses are reported, confirm that the following items are present in the relevant location (e.g. figure legend, table legend, main text, or Methods section).

n/a	Confirmed	
☐	☒	The exact sample size (*n*) for each experimental group/condition, given as a discrete number and unit of measurement
☐	☒	An indication of whether measurements were taken from distinct samples or whether the same sample was measured repeatedly
☐	☒	The statistical test(s) used AND whether they are one- or two-sided<br>*Only common tests should be described solely by name; describe more complex techniques in the Methods section.*
☐	☒	A description of all covariates tested
☐	☒	A description of any assumptions or corrections, such as tests of normality and adjustment for multiple comparisons
☐	☒	A full description of the statistics including central tendency (e.g. means) or other basic estimates (e.g. regression coefficient) AND variation (e.g. standard deviation) or associated estimates of uncertainty (e.g. confidence intervals)
☐	☒	For null hypothesis testing, the test statistic (e.g. *F*, *t*, *r*) with confidence intervals, effect sizes, degrees of freedom and *P* value noted<br>*Give P values as exact values whenever suitable.*
☒	☐	For Bayesian analysis, information on the choice of priors and Markov chain Monte Carlo settings
☒	☐	For hierarchical and complex designs, identification of the appropriate level for tests and full reporting of outcomes
☐	☒	Estimates of effect sizes (e.g. Cohen's *d*, Pearson's *r*), indicating how they were calculated
☐	☒	Clearly defined error bars<br>*State explicitly what error bars represent (e.g. SD, SE, CI)*

Our web collection on statistics for biologists may be useful.

Software and code

Policy information about availability of computer code

Data collection	We fully described in the Online Methods and Supplementary Note.
Data analysis	ALL softwares used in our study are shown in the Methods.

For manuscripts utilizing custom algorithms or software that are central to the research but not yet described in published literature, software must be made available to editors/reviewers upon request. We strongly encourage code deposition in a community repository (e.g. GitHub). See the Nature Research guidelines for submitting code & software for further information.

Data

Policy information about availability of data

All manuscripts must include a data availability statement. This statement should provide the following information, where applicable:

- Accession codes, unique identifiers, or web links for publicly available datasets
- A list of figures that have associated raw data
- A description of any restrictions on data availability

The summary statistics for the GWAS will be publicly available from the National Bioscience Database Center (NBDC) Human Database (https://humandbs.biosciencedbc.jp/en/) with the Data Set ID (hum0014) and from JENGER (http://jenger.riken.jp/en/). The genotype data employed in the GWAS are available from the Japanese Genotype-phenotype Archive (JGA; http://trace.ddbj.nig.ac.jp/jga/index_e.html) with accession codes JGAS00000000114 for the study

and JGAD00000000123 for the genotype data. The constructed reference panel for genotype imputation is also accessible through JGA upon request (accession code: JGAD00000000220). Height information can be provided by the Biobank Japan project upon a request (please see, https://biobankjp.org/english/index.html).

Field-specific reporting

Please select the best fit for your research. If you are not sure, read the appropriate sections before making your selection.

☒ Life sciences ☐ Behavioural & social sciences

For a reference copy of the document with all sections, see nature.com/authors/policies/ReportingSummary-flat.pdf

Life sciences

Study design

All studies must disclose on these points even when the disclosure is negative.

Sample size	We included the samples who were collected by five studies as much as possible.
Data exclusions	We excluded samples according to the standard quality controls (QC) procedure of GWAS. The details of QCs are described in the Methods.
Replication	To replicate the findings in GWAS, we used samples collected by four Japanese population-based cohorts. We basically considered P-values as the measurement of reproducibility; however, due to different sample sizes, we also evaluated the directional consistency and correlation in the effect sizes of identified variants between GWAS and replication study.
Randomization	Not applicable in our study design.
Blinding	Not applicable.

Materials & experimental systems

Policy information about availability of materials

n/a	Involved in the study	
☒	☐	Unique materials
☒	☐	Antibodies
☒	☐	Eukaryotic cell lines
☒	☐	Research animals
☐	☒	Human research participants

Human research participants

Policy information about studies involving human research participants

Population characteristics	This information is fully described in the Supplementary Note 2.

Method-specific reporting

n/a	Involved in the study	
☒	☐	ChIP-seq
☒	☐	Flow cytometry
☒	☐	Magnetic resonance imaging
