# Supplementary material for: Characterizing rare and low-frequency height-associated variants in the Japanese population
Source: Nat Commun. 2019 Sep 27;10:4393. doi: 10.1038/s41467-019-12276-5 (PMC6764965; doi:10.1038/s41467-019-12276-5)
Supplement: Supplementary file 3 — Description of Additional Supplementary Files [file 41467_2019_12276_MOESM3_ESM.pdf]

**Description of Additional Supplementary Files:**

Supplementary Data 1. Lead variants of the 355 autosomal loci.

Supplementary Data 2. Summary of the 363 height associated loci.

Supplementary Data 3. Additional 246 signals identified by conditional analysis.

Supplementary Data 4. Allele frequency spectrum of identified height associated variants across populations.

Supplementary Data 5. Replication and meta-analysis.

Supplementary Data 6. Associations of Japanese GWAS at reported variants in large-scale European GWAS.

Supplementary Data 7. Nonsynonymous variants in LD with height associated variants.

Supplementary Data 8. Gene-level associations by SKAT-O (MAF < 5%).

Supplementary Data 9. Single variant associations of nonsynonymous variants in *SLC27A3*.

Supplementary Data 10. Single variant associations of nonsynonymous variants in *CYP26B1*.

Supplementary Data 11. Significant gene-sets identified by PASCAL.
